# Influence of Modifying Additives on Resin-Bonded Diamond Tool and Single-Crystal Silicon Lapping Performance

**DOI:** 10.3390/ma19194163

**Published:** 2026-09-29

**Authors:** Tianchen Zhao, Zongding Bao, Sizhen Wu, Li-An Zhang, Wule Zhu, Luguang Guo, Kaiping Feng, Shuoshuo Qu

**Affiliations:** 1 The State Key Lab of Fluid Power and Mechatronic Systems, Zhejiang University, Hangzhou 310027, China; zhaotianchen1989@126.com (T.Z.); wulezhu@zju.edu.cn (W.Z.); 2 Zhejiang Key Laboratory of Intelligent Manufacturing for Aerodynamic Equipment, Quzhou University, Quzhou 324000, China; wusizhen@qzc.edu.cn (S.W.); glg7127@163.com (L.G.); fengkaiping11@126.com (K.F.); 3 Zhejiang Haina Semiconductor Co., Ltd., Quzhou 324000, China; zhanglian@hainasemi.cn; 4 School of Mechanical Engineering, ShanDong University, Jinan 250013, China; qushuoshuo@sdu.edu.cn

**Keywords:** single-crystal silicon, resin-bonded diamond tool, additives, orthogonal optimization

## Abstract

**Highlights:**

- A multifunctional resin-bonded diamond lapping tool was developed for single-crystal silicon.
- Polytetrafluoroethylene (PTFE) reduced interfacial friction and suppressed wafer temperature rise by 18.0%.
- CeO_2_ reduced surface roughness and machining-induced residual compressive stress.
- NH_4_HCO_3_ improved pore structure and sustained material removal during prolonged lapping.
- The balanced formulation achieved 8.46 nm Sa, 58.85 nm/min material-removal rate (MRR), a material removal-to-tool wear ratio (W-ratio) of 7.67 and a residual stress of −55.20 MPa.

**Abstract:**

To improve the interfacial tribological conditions, debris evacuation, and surface integrity in single-crystal silicon lapping using resin-bonded diamond tools, a multifunctional resin-bonded diamond tool incorporating lubricating, surface-quality-regulating, and pore-forming additives was developed. Single-factor experiments were conducted to evaluate the effects of graphite, MoS_2_, and Polytetrafluoroethylene (PTFE); CeO_2_, SiO_2_, and ZrO_2_; and NH_4_HCO_3_, NaHCO_3_, and NaCl on tool performance and silicon-surface quality. PTFE exhibited the best overall lubrication performance, reducing the temperature rise by 18.0% compared with the control. The addition of 1 wt.% CeO_2_ reduced the surface roughness Sa from 11.72 to 9.47 nm and decreased the residual compressive stress by approximately 86.5%. Moreover, 10 wt.% NH_4_HCO_3_ increased the apparent porosity from 6.76% to 32.42% and improved the sustainability of material removal. Based on the single-factor results, an L_9_ orthogonal experiment was performed using PTFE, CeO_2_, and NH_4_HCO_3_ as the control factors. Multi-response optimization identified a balanced formulation containing 1.5 wt.% PTFE, 1.0 wt.% CeO_2_, and 10 wt.% NH_4_HCO_3_. Validation experiments yielded a Sa of 8.46 nm, MRR = 58.85 nm/min, a material removal-to-tool wear ratio (W-ratio) of 7.67, and a residual stress of −55.20 MPa, demonstrating a favorable balance among surface quality, material-removal efficiency, and tool durability.

## 1. Introduction

Single-crystal silicon wafers are essential substrate materials for the fabrication of integrated circuits, power electronic devices, and optoelectronic components. Their surface topography, residual stress, and subsurface damage directly affect subsequent processes such as thin-film deposition and etching, as well as device reliability. With the increasing demand for low-defect surfaces in wafer lapping and polishing and highly integrated device manufacturing, achieving a machined surface with low roughness, minimal damage, and high consistency while maintaining sufficient material-removal efficiency has become an important issue in the ultra-precision machining of hard and brittle materials [1,2,3,4].

Single-crystal silicon is characterized by relatively high hardness, low fracture toughness, and a small brittle-to-ductile transition scale. Its material removal during lapping generally involves multiple deformation and removal modes, including elastic deformation, plastic flow, micro-cutting, and brittle fracture [5,6,7]. Gao et al. investigated the influence of surface integrity generated by preceding machining processes on subsequent polishing by comparing the surface morphology, subsurface damage, and subsequent chemical mechanical polishing behavior of single-crystal silicon wafers after grinding and lapping. They found that the surface textures and damaged-layer characteristics produced by different machining processes altered the actual contact conditions and material-removal behavior at the polishing interface [8]. To address the limitations of conventional loose-abrasive CMP (Chemical Mechanical Polishing) models in describing fixed-abrasive lapping, Chen et al. developed a material removal model for the fixed-abrasive lapping of ultra-hard and brittle materials by incorporating elastic–plastic contact, brittle-to-ductile transition, and abrasive compliance within the binder matrix. Their results indicated that binder hardness, abrasive size, abrasive population, and machining pressure jointly determine the penetration depth of abrasive grains and the resulting material-removal efficiency [9]. To reduce the mechanical damage readily induced by conventional rigid diamond abrasive tools, Gao et al. fabricated a lapping–polishing tool using ultrafine ZrO_2_ abrasives and a low-elastic-modulus nonwoven substrate. By investigating the effects of abrasive content and tool elastic modulus on the surface integrity of single-crystal silicon, they demonstrated that reducing tool stiffness and optimizing abrasive loading conditions could decrease subsurface damage while maintaining material-removal capability [10]. These studies indicate that abrasive penetration behavior, binder mechanical properties, and the condition of the working layer are critical factors governing the quality of fixed-abrasive machining.

To alleviate residual stress and near-surface damage induced by diamond-based mechanical machining, fixed-abrasive chemical mechanical machining has attracted increasing attention [11]. To address the severe mechanical damage associated with constant-feed diamond grinding and the difficulty of conventional CMP in simultaneously achieving high efficiency, Li et al. proposed a sequential thinning process combining constant-pressure diamond grinding with CeO_2_ fixed-abrasive chemical mechanical polishing. Their results showed that subsequent CeO_2_ fixed-abrasive polishing could effectively remove the grinding-induced damaged layer and reduce both the surface roughness and residual stress of silicon wafers [12]. On this basis, Li et al. investigated the effect of moisture on CeO_2_ fixed-abrasive polishing of single-crystal silicon by controlling environmental humidity. The results indicate that water molecules are important participants in the tribochemical reaction between CeO_2_ and the silicon surface, and that increasing environmental humidity promotes interfacial reactions and material removal [13]. Zhang et al. investigated the still-unclear mechanical role of relatively soft CeO_2_ abrasives during chemical mechanical grinding by conducting nanoscratch tests with CeO_2_ and diamond indenters, combined with numerical simulations of stress and damage evolution in silicon. They found that CeO_2_ abrasives exhibited limited direct mechanical cutting capability and that their contribution to chemical mechanical grinding depended more strongly on interfacial chemical reactions and the formation of a modified surface layer [14]. These findings suggest that chemically active abrasives such as CeO_2_ can promote the formation of a relatively easy-to-remove reaction layer on the silicon surface, thereby reducing the mechanical damage caused by the direct penetration of high-hardness abrasives.

The incorporation strategy of functional components at the machining interface has also gradually evolved from their addition to polishing slurries toward their immobilization within the abrasive tool itself [15]. To address the relatively low utilization efficiency of free abrasives in conventional polishing slurries, Bu et al. pretreated polishing pads with colloidal SiO_2_, allowing SiO_2_ particles to be pre-adsorbed onto the pad surface, and subsequently polished single-crystal silicon using an abrasive-free alkaline solution. Their results showed that the pre-adsorbed particles improved machining efficiency, process stability, and surface quality, with material removal resulting from the synergistic action of chemical modification of the silicon surface by the alkaline medium and the mechanical action of the adsorbed abrasives [16]. For the machining of similarly hard and brittle semiconductor materials, Xu et al. addressed the limited utilization of reactive species arising from the conventional incorporation of Fenton catalytic components in the liquid phase by introducing solid-phase catalytic components into a diamond gel polishing disk. They investigated solid-phase Fenton reaction-assisted polishing of SiC wafers and revealed a cyclic removal mechanism involving the formation of an oxidatively modified surface layer, mechanical removal by diamond abrasives, and subsequent exposure of a fresh surface [17]. This study demonstrates that directly integrating chemically active components into fixed-abrasive tools is a feasible approach for achieving synergistic regulation of mechanical removal and interfacial reactions.

Previous studies have elucidated the fundamental mechanisms of fixed-abrasive machining of single-crystal silicon from several perspectives, including abrasive penetration and brittle-to-ductile transition, binder or tool compliance, abrasive configuration at the machining interface, and chemical-reaction conditions [18,19,20]. However, most studies have focused on a single abrasive material, a single tool configuration, or an individual interfacial chemical factor, while mechanical machining and chemical mechanical polishing are often treated as separate processing stages [21,22,23]. For resin-bonded diamond abrasive tools in which diamond-induced mechanical removal is the dominant material removal mechanism, interfacial friction and heat generation, chip accommodation and self-sharpening associated with tool porosity, and the effect of chemically active components on silicon surface quality are inherently interrelated. Nevertheless, systematic studies integrating lubrication, surface-quality regulation, and pore-structure control within a single abrasive-tool system remain limited. In particular, the mechanisms and relative contributions of different modifying additives to surface roughness, material-removal rate, and abrasive-tool wear have not yet been fully clarified. Moreover, the competing relationships among surface quality, machining efficiency, and tool durability have rarely been systematically evaluated through multi-response analysis.

Accordingly, this study develops a multifunctional formulation for resin-bonded diamond abrasive tools by incorporating solid lubricants, surface-quality modifiers, and pore-forming agents. Based on single-factor screening experiments, Polytetrafluoroethylene (PTFE), CeO_2_, and NH_4_HCO_3_ were selected for a three-factor, three-level L_9_ orthogonal experiment. Signal-to-noise ratio analysis and analysis of variance were employed to investigate the effects and relative contributions of the contents of the three modifying additives to surface roughness Sa, material-removal rate (MRR), and material removal-to-tool wear ratio (G-ratio). A formulation validation experiment was subsequently conducted to evaluate the multi-response optimization result. By integrating interfacial lubrication, pore-structure construction, and potential chemical assistance within a single resin-bonded diamond lapping tool system, this study establishes quantitative relationships between the three categories of modifying additives and the corresponding machining responses.

## 2. Experimental Materials and Methods

### 2.1. Machining Principle and Methods

In this study, single-crystal silicon wafers were processed by fixed-abrasive lapping using resin-bonded diamond tools, in which the abrasive grains are held within a resin matrix. Under the applied normal load and relative motion between the tool and workpiece, diamond grains exposed at the tool surface remove small amounts of material through indentation, micro-cutting, and plowing. Lapping was performed at a low rotational speed under a constant normal load for a controlled duration. Although the process is dominated by two-body abrasive wear and shares certain microscopic material removal mechanisms with grinding, it is referred to here as fixed-abrasive lapping based on the equipment, contact configuration, kinematics, and loading method. This designation distinguishes the process from traditional loose-abrasive lapping and conventional wheel grinding [9,24].

**Figure 1:**
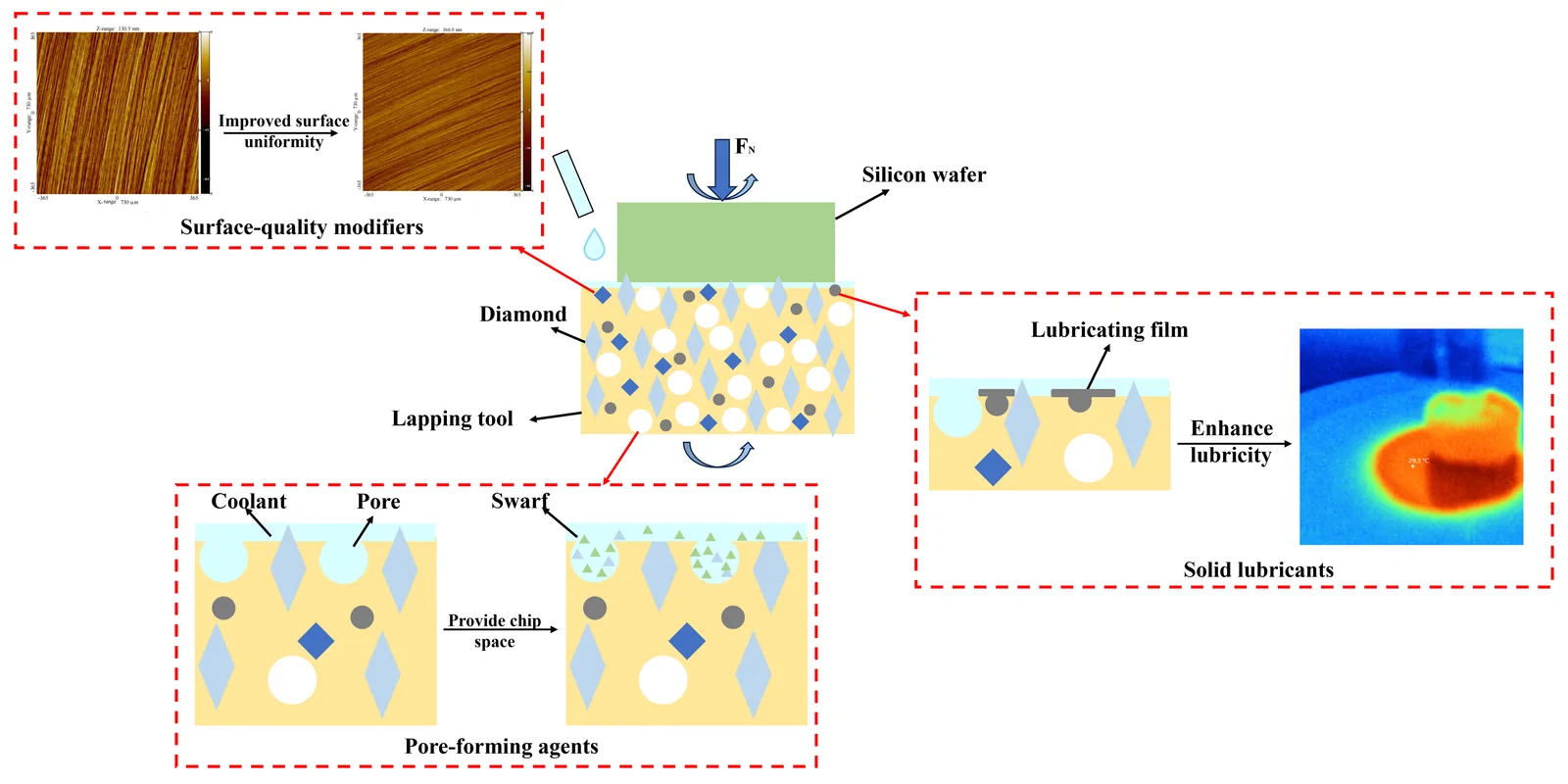
Schematic illustration of the machining principle of single-crystal silicon lapping.

Figure 1 schematically illustrates the principle of single-crystal silicon lapping, in which material removal is governed primarily by the mechanical action of diamond grains [25,26]. When the lapping fluid enters the contact zone, pores within the tool can provide pathways for fluid transport and space for debris accommodation, while facilitating the release of dulled grains and the exposure of fresh grains. Solid lubricants can reduce interfacial shear resistance and adhesion. Surface-quality modifiers may promote the formation of a softened layer on the silicon surface through interfacial chemical reactions, making the layer more readily removable and improving the uniformity of the machined surface [11,27,28]. In this system, the modifying additives primarily improve the stability of material removal and surface quality through lubrication, pore-structure control, and chemical assistance.

The single-crystal silicon wafers used in this study had a <100> orientation, with the (100) plane as the surface subjected to lapping. During processing, the instantaneous sliding directions of the abrasive grains spanned multiple crystallographic directions within the (100) plane. No fixed alignment was imposed between a single cutting direction and a specific crystallographic direction. The results therefore reflect the overall material-removal behavior of the silicon (100) surface under multidirectional abrasive action.

**Figure 2:**
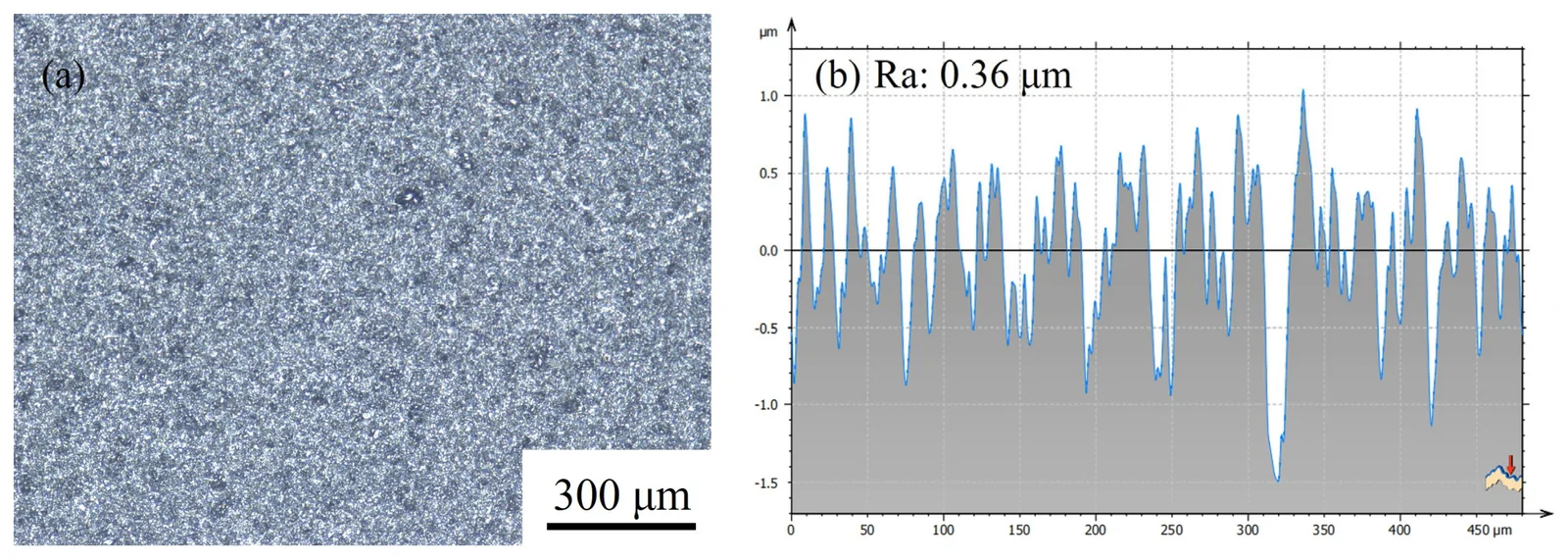
Initial surface morphology of the single-crystal silicon wafer specimen: (**a**) surface morphology; (**b**) Ra.

All wafers underwent the same surface-pretreatment procedure. The wafer surfaces were pre-lapped using a #2200 diamond slurry (Zhecheng Hongguang Diamond Technology Co., Ltd., Zhecheng, China) and subsequently characterized using a surface roughness profilometer to establish their initial surface condition. As shown in Figure 2a, the pre-lapped surface exhibited numerous pits, abrasive scratches, and localized fracture damage, which are characteristic of a coarsely lapped surface. The roughness measurements in Figure 2b indicate an initial Ra of approximately 0.36 μm.

**Figure 3:**
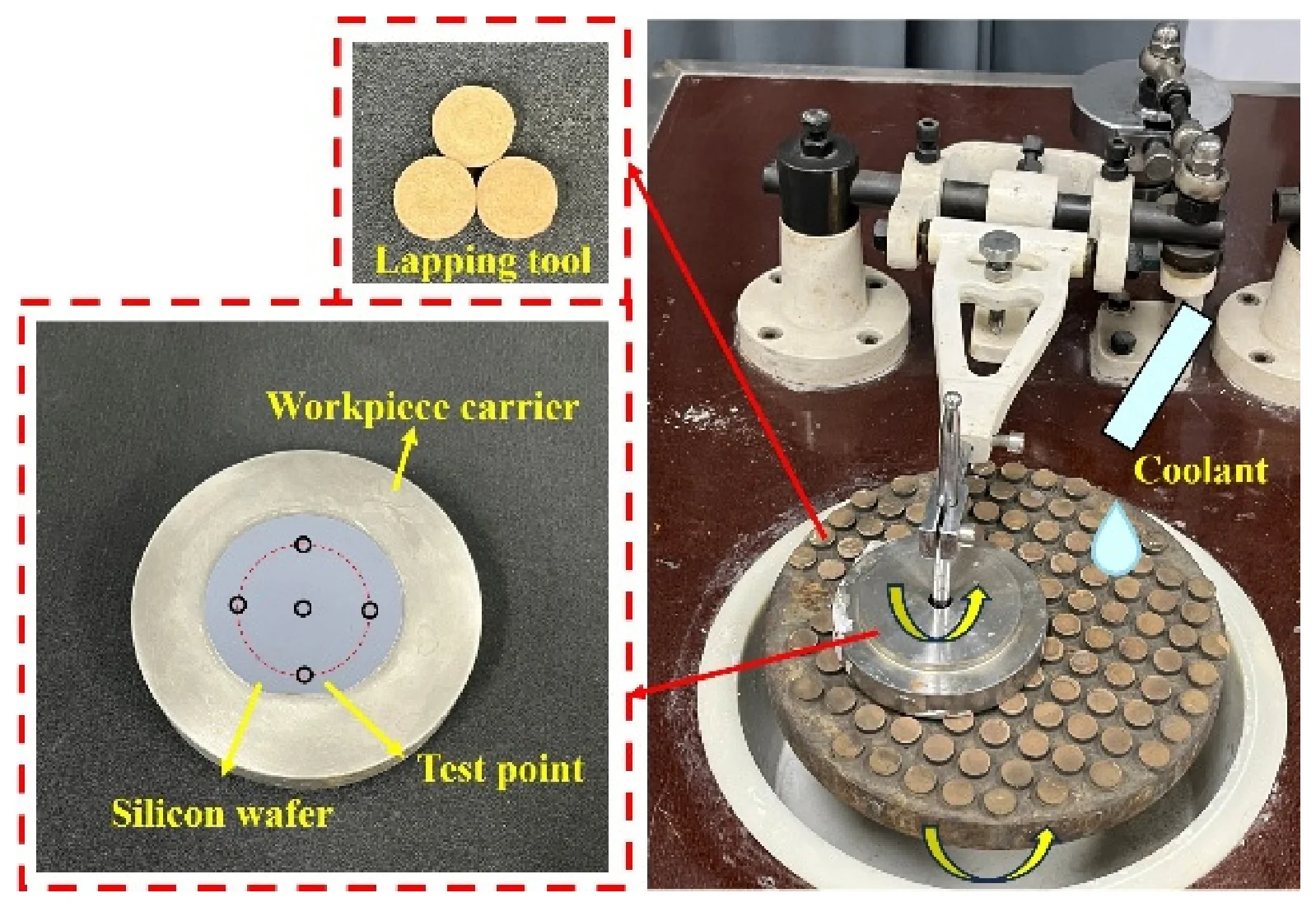
Experimental setup.

Figure 3 shows the experimental setup for the lapping experiments. Experiments were performed using a four-axis lapping–polishing machine (Xibin Optoelectronics Co., Ltd., Yancheng, China). The wafer was attached to the workpiece carrier and placed in contact with the lapping tool for lapping. The rotational speed of the worktable spindle was set to 60 rpm, and a load of 1 kg was applied to the wafer. Purified water was supplied as the coolant at a flow rate of 20 mL/min.

### 2.2. Preparation of Resin-Bonded Diamond Tools

The resin-bonded diamond tools consisted primarily of diamond abrasives, a resin binder, fillers, and modifying additives. Phenolic resin was used as the binder. The nominal particle sizes were 7 μm for the solid lubricants [graphite (Shenzhen Xindun New Material Technology Co., Ltd., Shenzhen, China), MoS_2_ (Qingdao Zhongye New Material Technology Co., Ltd., Qingdao, China), PTFE (Shandong Feihong Plastic Products Co., Ltd., Jining, China)], 500 nm for the surface-quality modifiers [CeO_2_, SiO_2_, and ZrO_2_ (Qingdao Zhongye New Material Technology Co., Ltd., Qingdao, China)], and 75 μm for the pore-forming agents [NH_4_HCO_3_, NaHCO_3_, and NaCl (Tianjin Huasheng New Materials Technology Co., Ltd., Tianjin, China)]. The basic tool formulation is presented in Table 1.

The tools were compacted at 500 MPa and subsequently cured at 160 °C for 2 h. The resulting cylindrical tools, each 12 mm in diameter and 5 mm thick (Figure 3), were bonded in a staggered arrangement to a lapping base plate with a diameter of 200 mm. The working surfaces of the mounted tools were then ground flat using a high-precision grinding machine (SLG 80, Schneider GmbH and Co. KG, Fronhausen, Germany). The grinding machine has a feed accuracy of 1 μm, ensuring good flatness of the ground tool surface.

To reduce the effects of silicon debris adhesion, surface loading, and localized glazing on subsequent experiments, the working surfaces of the tools were dressed with a #80 green silicon carbide grinding wheel after each experiment. The tools were then ultrasonically cleaned to remove residual debris. This dressing and cleaning procedure restored the exposure of diamond grains and ensured consistent and reproducible initial working surface conditions across the lapping experiments.

### 2.3. Performance Testing and Characterization Methods

The formulation listed in Table 1 was used as the base formulation for all comparative experiments. For the solid-lubricant study, PTFE, graphite, and MoS_2_ were individually incorporated into the base formulation at a content of 1 wt.% to prepare three groups of solid-lubricant-containing lapping tools. A tool prepared using the base formulation without the addition of a solid lubricant served as the control. The four groups of tools were subsequently evaluated under identical conditions to determine the effects of the solid lubricants on the interfacial friction and thermal behavior of the lapping tools.

For the surface-quality-modifier study, CeO_2_, SiO_2_, and ZrO_2_ were individually incorporated into the base formulation at a content of 1 wt.%. The three modified tools and the corresponding control tool were used to lap single-crystal silicon wafers under identical processing conditions. After lapping, the three-dimensional surface topography of each wafer was characterized using white-light interferometry. The Raman response of the lapped silicon surface was further examined using micro-Raman spectroscopy. Each Raman spectrum was fitted with a Gaussian function to determine the characteristic peak position, which was subsequently used to evaluate the Raman-derived equivalent residual stress.

For the pore-former study, NH_4_HCO_3_, NaHCO_3_, and NaCl were individually incorporated into the base formulation at a content of 10 wt.%, and a tool prepared without a pore-forming agent served as the control. NH_4_HCO_3_ underwent thermal decomposition during curing and acted as a fugitive pore former. NaHCO_3_ thermally decomposed to form Na_2_CO_3_, H_2_O, and CO_2_, leaving Na_2_CO_3_ within the tool, whereas NaCl remained stable under the curing conditions. After curing, the tools containing NaHCO_3_ and NaCl were washed with water to dissolve the residual Na_2_CO_3_ and NaCl, respectively, thereby generating open pores. The apparent porosity of the resulting tools was subsequently measured, and their material-removal rates were monitored as a function of lapping time over a period of 0–60 min [29,30,31].

Four single-crystal silicon wafers with identical specifications and surface pretreatment were used for the temperature-rise tests, one each for the control tool and the tools containing graphite, MoS_2_, and PTFE. Each wafer was used for only one independent temperature-rise test with its corresponding tool. This procedure prevented cumulative material loss and changes in wafer heat capacity caused by repeated lapping from affecting comparisons among the tools. The wafer surface temperature was measured at a fixed location using a Ti100 infrared thermal imager (Fluke Corporation, Everett, WA, USA), and the temperature was recorded at intervals of 0.5 min for a total duration of 10 min. Friction and wear tests were conducted using an HT-1000 high-temperature tribometer (Lanzhou Zhongke Kaihua Technology Development Co., Ltd., Lanzhou, China) to measure the friction coefficient of the lapping tool surface. The tests were performed at room temperature using a ball-on-disk contact configuration. A Si_3_N_4_ ball with a diameter of 3.969 mm was used as the counterface. The sliding velocity, wear-track radius, normal load, and test duration were set to 2 m/s, 3 mm, 3.5 N, and 5 min, respectively.

The apparent porosity of the samples was determined using the Archimedes principle:

The total specimen volume comprises the volumes of the solid phase, open pores, and closed pores, and apparent porosity is defined as the fraction of this total volume occupied by open pores. A specimen with an initial dry mass of *m*_0_ was placed in a beaker, and purified water was added until the specimen was fully immersed. The beaker was heated on a hot plate, and the water was maintained at boiling for 30 min before heating was discontinued. The water-saturated specimen was then placed in the suspension basket of a density balance, and its apparent mass while immersed in water was recorded as *m*_1_. The specimen was subsequently removed, gently blotted with a damp cloth to remove surface water, and weighed in air to obtain its saturated mass, *m*_2_.

The bulk density, *ρ*_b_, is calculated as

(1) $$\rho_{b}=\frac{m_{0}\rho_{1}}{m_{2}-m_{1}}$$

where *ρ*_1_ is the density of the immersion liquid at the measurement temperature (g/cm^3^), *m*_0_ is the dry specimen mass, *m*_1_ is the apparent mass of the saturated specimen immersed in the liquid, and *m*_2_ is its saturated mass measured in air.

The apparent porosity, *P*_a_, is calculated as

(2) $$P_{a}=\frac{\rho_{a}-\rho_{b}}{\rho_{a}}\times 100\%$$

where *ρ*_a_ is the apparent solid density, given by

(3) $$\rho_{a}=\frac{m_{0}\rho_{1}}{m_{0}-m_{1}}$$

The material-removal rate (MRR) and material-removal-to-tool-wear ratio (W-ratio) were determined from the mass changes in the silicon specimens and lapping tools measured using an electronic analytical balance with an accuracy of 0.1 mg. In wafer lapping, MRR is commonly expressed as the equivalent average thickness removed per unit time [32]. It is calculated as

(4) $$\mathrm{MRR}=\frac{\Delta m}{\mathrm{\rho st}}$$

where Δ*m* is the mass of silicon removed during lapping, *ρ* is the density of single-crystal silicon, taken as 2.33 g/cm^3^, *s* is the lapped surface area, and *t* is the lapping duration. For the 2-inch silicon wafers used in this study, *s* was taken as 20.1285 cm^2^. With Δ*m* expressed in g and *t* in min, the calculated rate is in cm/min; multiplication by 10^7^ converts it to nm/min.

Because tool wear was nonuniform during lapping, the wear depth at a single location or a limited number of profile measurements could not adequately represent the overall material loss from the tool. Tool wear was therefore evaluated gravimetrically. Before and after each lapping experiment, the tools were cleaned using the same procedure, dried to constant mass, and weighed. The mass-based W-ratio is calculated as [33]:

(5) $$W=\frac{m_{w1}-m_{w2}}{m_{s1}-m_{s2}}$$

where *m*_w1_ and *m*_w2_ are the masses of the silicon workpiece before and after lapping, respectively, and *m*_s1_ and *m*_s2_ are the corresponding masses of the lapping tool.

The working surfaces of the dressed tools were examined using a digital microscope with an extended depth of field (VHX-2000C, Keyence Corporation, Osaka, Japan) at 500× magnification.

The surface quality of the lapped silicon wafers was characterized using a white-light interferometer (MicroXAM, KLA-Tencor, Milpitas, CA, USA) and a surface profilometer (PGI NOVUS, Taylor Hobson, Leicester, UK). Measurements were taken at five locations on each wafer: the center and four points on a circle 30 mm in diameter, as shown in Figure 3. For the Ra measurements, the stylus was scanned perpendicular to the local dominant surface, in accordance with ISO 21920 [34]. A diamond stylus with a tip radius of 2 μm, an included cone angle of 60°, and a contact force of 80 mgf was used. The measurement settings were *λ_c_* = 0.08 mm, *λ_s_* = 2.5 μm, *l_e_* = 0.4 mm, and a scanning speed of 0.1 mm/s. The structural characteristics of the silicon wafers were analyzed using an RTSmini-301MS532-SMS micro-Raman spectrometer (Beijing Zolix Instruments Co., Ltd., Beijing, China) with a laser wavelength of 532 nm, a laser power of 100 mW, and a measurement range of 90–9000 cm^−1^. The residual stress on the lapped silicon surface was evaluated from the Raman peak shift. For strain-free single-crystal silicon, a sharp characteristic Raman peak appears at approximately 520.7 cm^−1^. When silicon is subjected to uniaxial stress, the position of this Raman peak shifts accordingly [35]. The uniaxial residual stress is calculated using Equation (6):

*σ* = −434 Δ*ω* (6)

where σ is the residual stress in MPa and Δω is the Raman peak shift in cm^−1^, defined as the measured peak position minus the stress-free reference value of 520.7 cm^−1^.

## 3. Results and Discussion

### 3.1. Effects of Solid Lubricants on the Interfacial Friction and Thermal Behavior During Lapping

**Figure 4:**
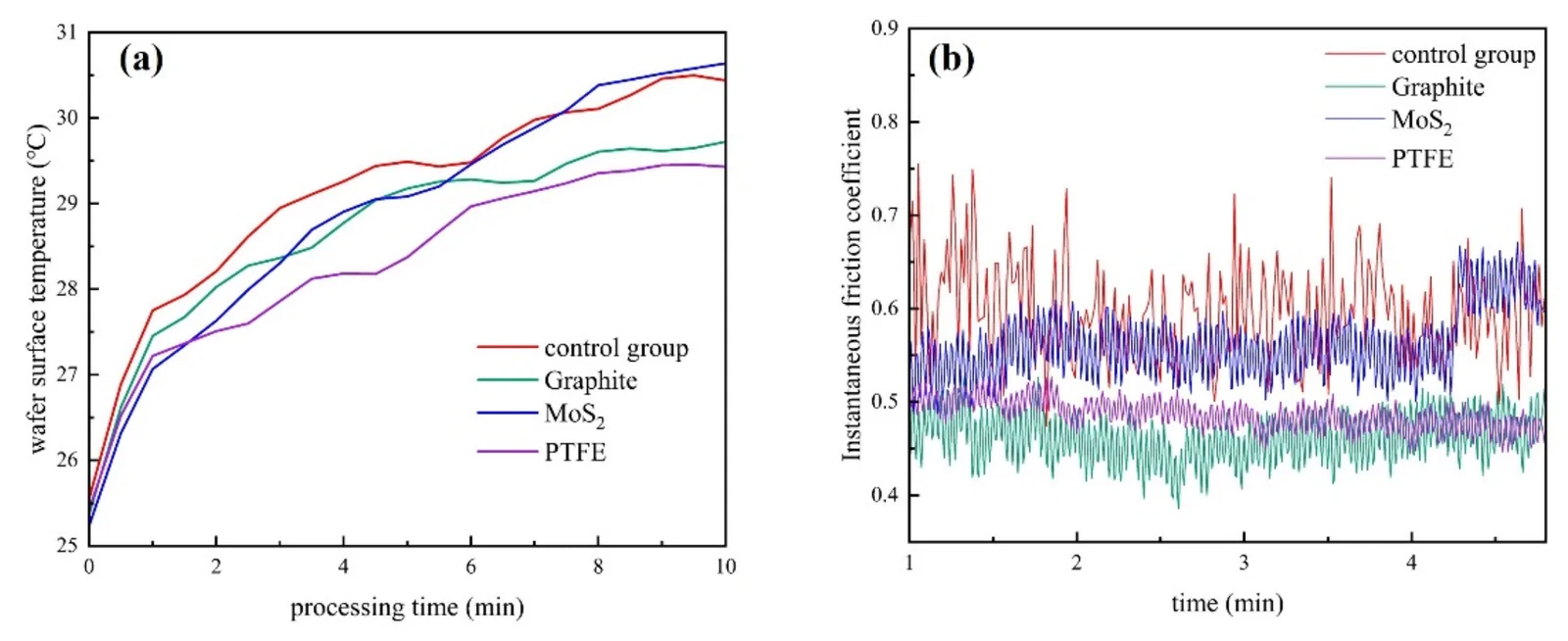
(**a**) Wafer surface temperature and (**b**) instantaneous coefficient of friction for lapping tools containing different solid lubricants.

The results of the machining temperature measurements are shown in Figure 4a. The initial temperatures of the four groups were approximately 25.2–25.5 °C. As the lapping time increased, the wafer surface temperature in all groups increased rapidly at first, followed by a slower increase and gradual stabilization. At the initial stage of lapping, the lapping tool first came into contact with the silicon wafer, and the heat generated by abrasive cutting, binder friction, and sliding of machining debris could not be dissipated sufficiently and promptly into the lapping tool, the wafer, and the surrounding environment, resulting in a rapid increase in wafer surface temperature. As lapping proceeded, the contact interface gradually entered a relatively stable running-in state. Heat generation and heat dissipation at the interface gradually approached a balance, leading to a reduced rate of temperature rise. The short plateau or slow-increase stage observed at approximately 4–6 min may be associated with the running-in of the lapping tool surface, redistribution of machining debris, and the temporary dynamic equilibrium between heat generation and heat dissipation.

The PTFE group maintained a relatively low surface temperature during most of the lapping process. After 10 min, the temperature rise was 4.1 °C, which was 18.0% lower than that of the control group, indicating the most pronounced suppression of temperature rise among the four groups. Owing to its low surface energy and low shear strength, PTFE may form a low-shear-strength transfer layer between the lapping tool and the silicon wafer during friction, thereby reducing adhesion and sliding interactions among the binder, machining debris, and silicon surface and consequently decreasing interfacial frictional heat generation. Between 6 and 10 min, the temperature of the PTFE group increased by only 0.5 °C, indicating that its lubricating effect remained relatively stable over time. The graphite group exhibited a temperature rise of 4.5 °C after 10 min, representing a 10.0% reduction compared with the control group, and therefore also showed a certain cooling effect. The layered crystal structure of graphite facilitates interlayer sliding under shear and may promote the formation of a lubricating film at the contact interface, thereby reducing frictional resistance and heat generation. However, the reduction in temperature rise was smaller than that of the PTFE group, suggesting that under the present formulation and lapping conditions, the continuity or stability of the graphite lubricating film may have been inferior to that of the PTFE transfer layer.

The surface temperature of the MoS_2_ group was lower than that of the control group during the early stage of lapping, suggesting that MoS_2_ may provide a certain lubricating effect during the initial running-in period. However, its temperature continued to increase after 6 min and exceeded that of the control group at approximately 8 min. After 10 min, the temperature rise reached 5.5 °C, the highest among the four groups. This result indicates that 1 wt.% MoS_2_ was unable to form a stable lubricating film and therefore failed to provide sustained lubrication. This tendency is consistent with the increase in the coefficient of friction observed for the MoS_2_ group during the later stage of the friction and wear test, suggesting that the interfacial lubrication state gradually became unstable with increasing lapping time.

Figure 4b presents the variations in the instantaneous coefficient of friction for lapping tools containing different solid lubricants, and the corresponding average coefficients of friction are listed in Table 2. Compared with the control group, the addition of graphite, MoS_2_, and PTFE reduced the average coefficient of friction by 22.7%, 5.9%, and 18.5%, respectively. These results indicate that all three solid lubricants improved the tribological performance of the lapping tools to varying degrees. Graphite exhibited the most pronounced friction-reducing effect, followed by PTFE, whereas MoS_2_ showed only a limited reduction in friction.

Although the average coefficient of friction of the PTFE group was higher than that of the graphite group, the PTFE group exhibited a lower wafer temperature rise. This difference can be attributed to the fact that the macroscopic temperature rise in the silicon wafer is not determined solely by the coefficient of friction, but is also influenced by factors such as the real contact area, material-removal behavior, and the movement of machining debris. In addition, different counterface materials were used in the two tests: a Si_3_N_4_ ball was used in the friction and wear test, whereas a single-crystal silicon wafer was used in the machining temperature test. Therefore, a strict one-to-one correspondence between the friction coefficient and wafer temperature rise should not be expected. Graphite exhibited the lowest average coefficient of friction and therefore showed strong direct friction-reducing capability. PTFE, although exhibiting a slightly higher coefficient of friction than graphite, provided a relatively low friction coefficient, a more stable frictional response, and the lowest wafer temperature rise. Accordingly, PTFE exhibited the best overall lubrication performance and was selected as the solid lubricant for the orthogonal experiments.

### 3.2. Effect of Surface-Quality Modifiers on the Machining Quality of Silicon Wafers

**Figure 5:**
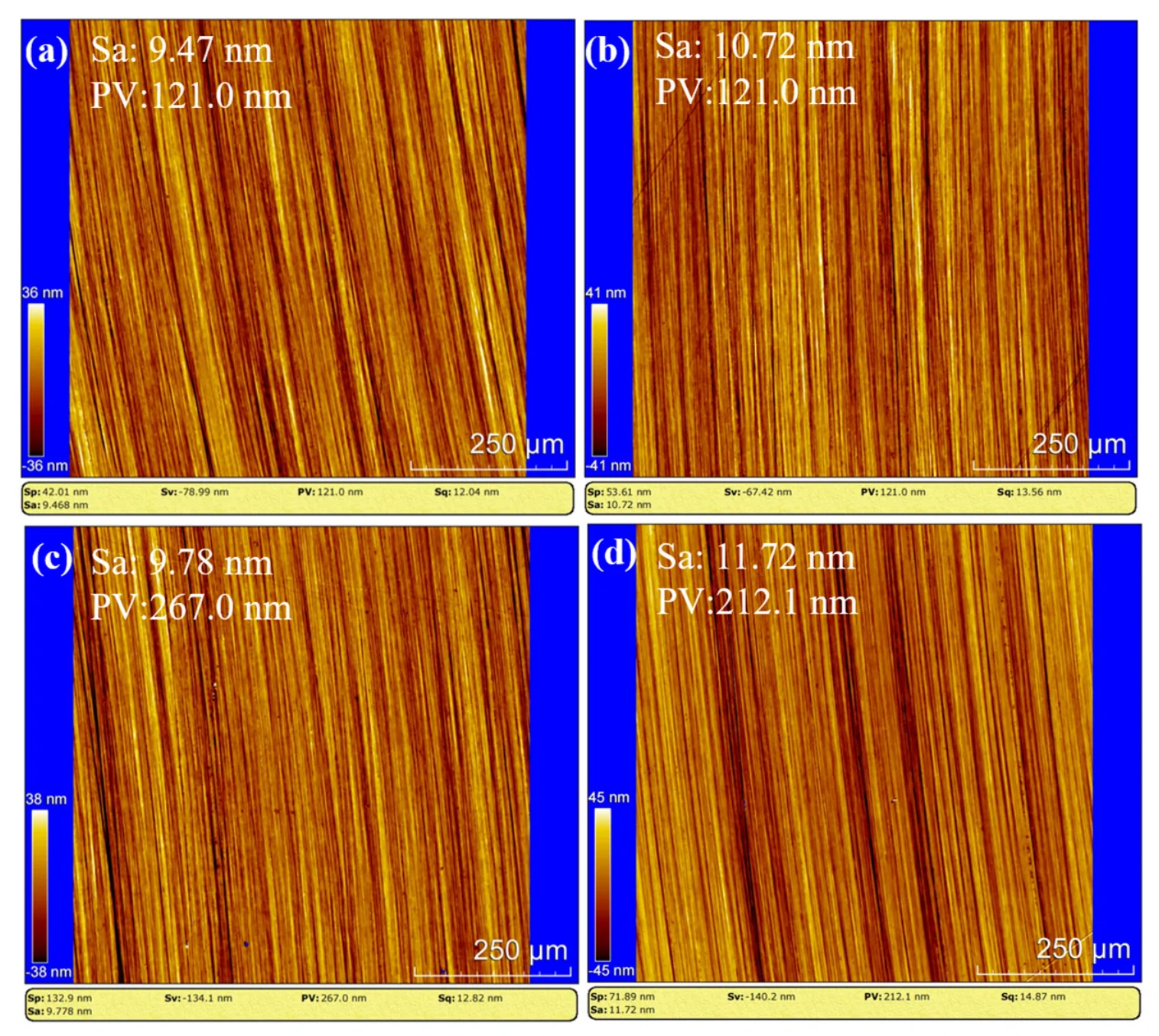
White-light interferometric surface topographies of silicon wafers lapped using tools containing different surface-quality modifiers: (**a**) CeO_2_; (**b**) SiO_2_; (**c**) ZrO_2_; (**d**) control group.

**Figure 6:**
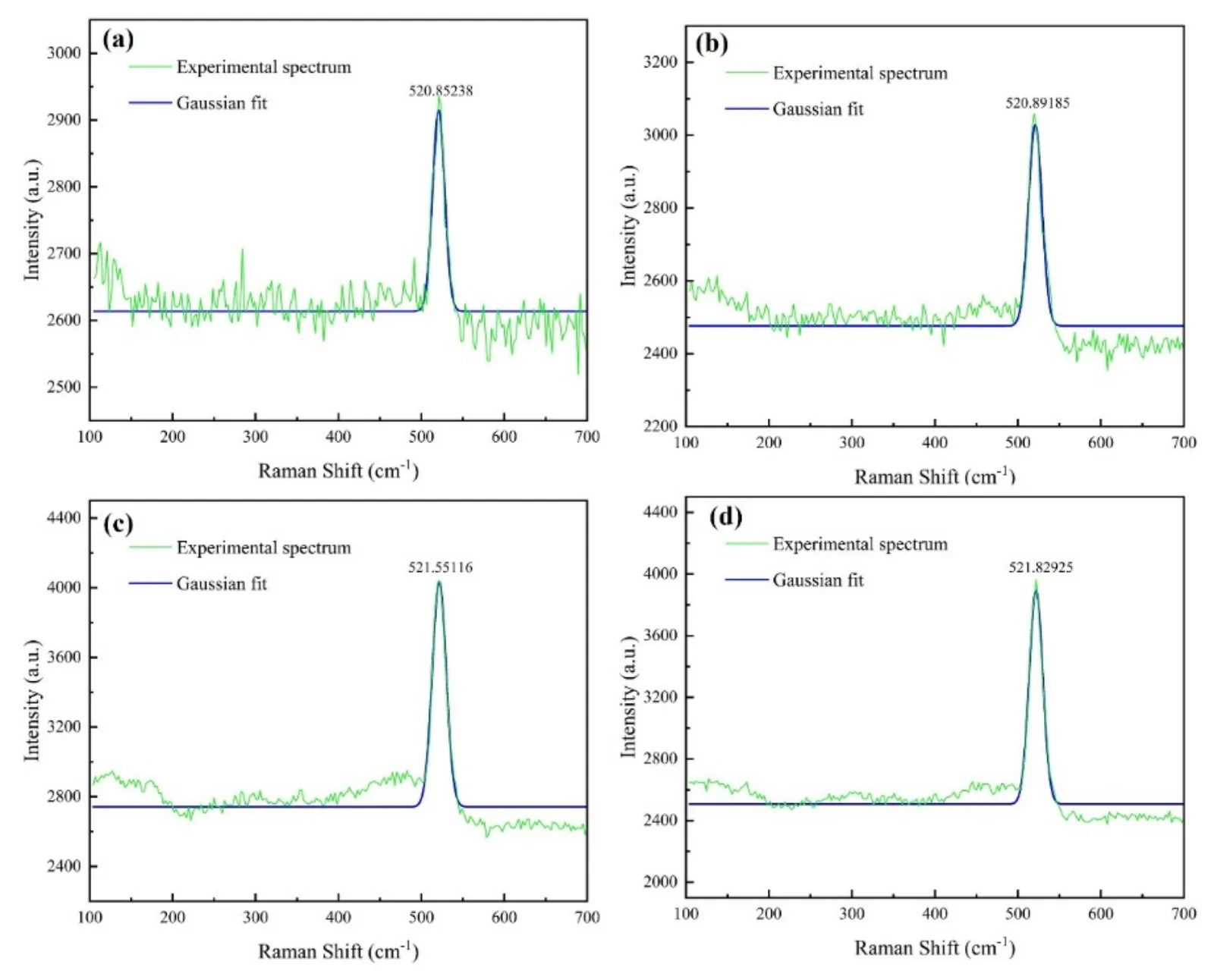
Raman spectra and Gaussian fitting results of silicon wafers lapped using tools containing different surface-quality modifiers: (**a**) CeO_2_; (**b**) SiO_2_; (**c**) ZrO_2_; (**d**) control group.

The three-dimensional surface topographies obtained using the different surface-quality modifiers are presented in Figure 5. The corresponding Raman spectra and Gaussian fitting results are shown in Figure 6 and summarized in Table 3, respectively. The surfaces of all four groups exhibited parallel grooves distributed along the machining direction, indicating that the lapped surfaces were still predominantly governed by the plowing and sliding actions of diamond abrasives. However, noticeable differences were observed in groove uniformity and local height variation among the surfaces machined with different modifiers. The control group exhibited a Sa of 11.72 nm and a PV of 212.1 nm, with relatively pronounced directional grooves and local surface-height fluctuations. After the addition of CeO_2_, Sa decreased to 9.47 nm, representing a reduction of 19.2% compared with the control group, while PV decreased to 121.0 nm, corresponding to a reduction of 42.9%. The CeO_2_ group exhibited the lowest Sa together with a relatively low PV, indicating that CeO_2_ not only improved the overall surface smoothness of the silicon wafer but also effectively suppressed local deep scratches or abnormal protrusions.

For the SiO_2_ group, Sa and PV were 10.72 nm and 121.0 nm, respectively, corresponding to reductions of 8.5% and 42.9% relative to the control group. Although the improvement in Sa was smaller than that obtained with CeO_2_, the PV value was identical to that of the CeO_2_ group. This result suggests that SiO_2_ was effective in suppressing severe local surface defects, whereas its improvement in overall microscale roughness was relatively limited. The ZrO_2_ group exhibited a Sa of 9.78 nm, which was 16.6% lower than that of the control group, whereas the PV increased to 267.0 nm, representing an increase of 25.9%. Thus, although the ZrO_2_-modified lapping tool produced a relatively low average surface roughness, local defects such as deeper grooves, particle indentation, or material spalling were still present. The relatively high hardness of ZrO_2_ and local asperities arising from particle agglomeration may increase the local contact load and plowing action, thereby resulting in a larger peak-to-valley height difference.

Figure 6 presents the Raman spectra and corresponding Gaussian fitting results of the silicon wafer surfaces machined using the different modified lapping tools. All four groups exhibited the characteristic Raman peak of single-crystal silicon near 520 cm^−1^, and the fitted curves generally agreed well with the experimental peak profiles. Relative to the reference peak position of 520.7 cm^−1^ for stress-free single-crystal silicon, the characteristic peaks of all groups shifted to higher wavenumbers to varying degrees, indicating the presence of residual compressive stress on the machined surfaces.

The Raman peak of the control group was located at 521.82925 cm^−1^, corresponding to a residual stress of −490.09 MPa, which represented the highest compressive stress among the four groups. This result suggests that, in the absence of surface-quality modifiers, mechanical compression and plowing by abrasive grains, together with thermo-mechanical effects during lapping, may induce relatively severe lattice compression and residual deformation. The CeO_2_ group exhibited a Raman peak at 520.85238 cm^−1^, which was the closest to the stress-free reference position, corresponding to a residual stress of −66.13 MPa. Compared with the control group, the magnitude of compressive stress was reduced by approximately 86.5%, indicating that CeO_2_ effectively alleviated machining-induced lattice distortion and residual stress accumulation.

For the SiO_2_ group, the Raman peak was located at 520.89185 cm^−1^, corresponding to a residual stress of −83.26 MPa, which was approximately 83.0% lower in magnitude than that of the control group. This result indicates that SiO_2_ also effectively reduced the residual compressive stress on the lapped surface, although the degree of stress reduction was slightly lower than that obtained with CeO_2_. The ZrO_2_ group exhibited a Raman peak at 521.55116 cm^−1^, corresponding to a residual stress of −369.40 MPa. Although the magnitude of compressive stress was reduced by approximately 24.6% compared with the control group, it remained substantially higher than those of the CeO_2_ and SiO_2_ groups. Combined with its relatively high PV value, this result suggests that although the addition of ZrO_2_ improved the average roughness in some regions, locally intensified mechanical compression, plowing, or brittle damage may still have caused pronounced lattice distortion.

### 3.3. Effects of Pore-Forming Agents on the Pore Structure and Material-Removal Performance of Lapping Tools

**Figure 7:**
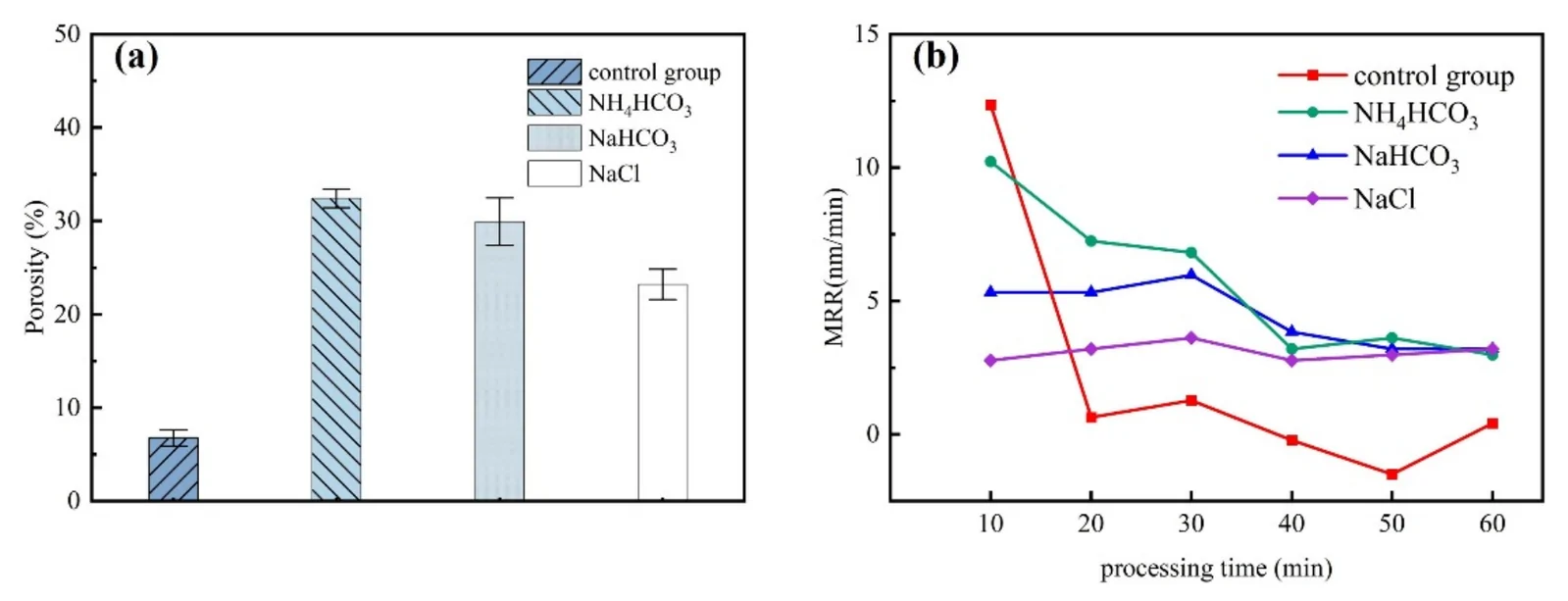
(**a**) Apparent porosity and (**b**) material-removal rate as a function of lapping time for tools containing different pore-forming agents.

Figure 7a shows the apparent porosity of the corresponding lapping tools, while Figure 7b presents the variation in material-removal rate (MRR) with lapping time from 0 to 60 min for the lapping tools containing different pore-forming agents.

As shown in Figure 7a, the incorporation of pore-forming agents markedly increased the apparent porosity of the lapping tools. The control group exhibited an average apparent porosity of only 6.76%, indicating that a relatively dense structure was formed after curing of the basic formulation. In contrast, the average apparent porosities of the NH_4_HCO_3_, NaHCO_3_, and NaCl groups increased to 32.42%, 29.93%, and 23.22%, respectively. The NH_4_HCO_3_ and NaHCO_3_ groups exhibited similarly high porosity levels, whereas the NaCl group, although showing an evident pore-forming effect, exhibited a lower porosity than the other two groups.

The differences in the pore structures produced by the different pore-forming agents were mainly associated with their physicochemical behavior during curing. Upon heating, NH_4_HCO_3_ decomposes into NH_3_, CO_2_, and H_2_O. The escape of these gaseous products generates abundant pores within the lapping tool, while leaving essentially no solid residue, resulting in the highest apparent porosity. Although NaHCO_3_ releases CO_2_ and H_2_O during thermal decomposition, solid Na_2_CO_3_ is simultaneously formed. Subsequent water washing can dissolve and remove part of the residual Na_2_CO_3_, thereby generating additional pores. However, Na_2_CO_3_ completely encapsulated by the resin binder may not be sufficiently dissolved and removed, resulting in nonuniform pore formation. This may also account for the relatively large error range observed for this group. For the NaCl group, because NaCl does not decompose at the curing temperature, pore formation relies entirely on its dissolution during post-curing water washing. Only NaCl particles connected to the lapping tool surface or to existing interconnected channels can be effectively removed, whereas particles encapsulated within the resin matrix may remain or form closed regions. Consequently, the final apparent porosity of the NaCl group was relatively low.

As shown in Figure 7b, the MRRs of the different lapping tools varied to different extents with increasing lapping time. At the initial stage of lapping, i.e., at 10 min, the control group exhibited the highest MRR of approximately 12.27 nm/min, whereas the MRRs of the NH_4_HCO_3_, NaHCO_3_, and NaCl groups were approximately 10.23, 5.33, and 2.77 nm/min, respectively. The relatively high initial MRR of the control group may be attributed to the larger real contact area of the dense lapping tool and the relatively sharp diamond abrasives on its initial working surface, resulting in a larger number of effective abrasives participating in material removal. However, after 10 min of lapping, the MRR of the control group rapidly decreased to nearly zero and exhibited apparent negative values at 40–50 min, indicating that its material-removal capability could not be sustained. Owing to the lack of sufficient chip-accommodation space, silicon debris, resin wear debris, and fractured abrasive particles generated during lapping readily accumulated on the lapping tool surface, leading to tool loading and surface dulling and thereby substantially reducing the cutting effectiveness of the active diamond abrasives.

Compared with the control group, all three porous lapping tools exhibited more stable material-removal behavior. The NH_4_HCO_3_ group maintained a relatively high MRR during 10–30 min, after which it gradually decreased and stabilized at approximately 2.9–3.2 nm/min during 40–60 min. The MRR of the NaHCO_3_ group remained at approximately 5.6–6.0 nm/min during the first 30 min and decreased to approximately 3 nm/min after 40 min. The NaCl group exhibited the lowest initial MRR and remained relatively stable during the subsequent lapping period. These results indicate that the pore structure provides space for the accommodation and evacuation of coolant and machining debris, alleviates loading of the lapping tool surface, and facilitates the shedding of worn abrasives and the exposure of fresh cutting edges, thereby improving the sustainability of the material-removal process.

Although the NH_4_HCO_3_ and NaHCO_3_ groups exhibited similar apparent porosities, the former showed a substantially higher MRR during the initial stage of lapping. This indicates that the material-removal performance of the lapping tool is not governed solely by its overall porosity, but is also affected by factors such as pore distribution uniformity and inorganic residues. If the Na_2_CO_3_ generated by NaHCO_3_ decomposition is not completely removed, it may occupy part of the pore volume and reduce pore interconnectivity, thereby weakening the effective chip-accommodation capability of the pore structure. After approximately 40 min of lapping, the MRRs of the three porous lapping tools gradually converged. This suggests that, as the initially exposed abrasives progressively wore and the surface morphology of the lapping tools approached a relatively stable state, the material-removal process gradually reached a dynamic equilibrium, and the influence of the different pore-forming agents on the instantaneous removal capability became less pronounced.

**Figure 8:**
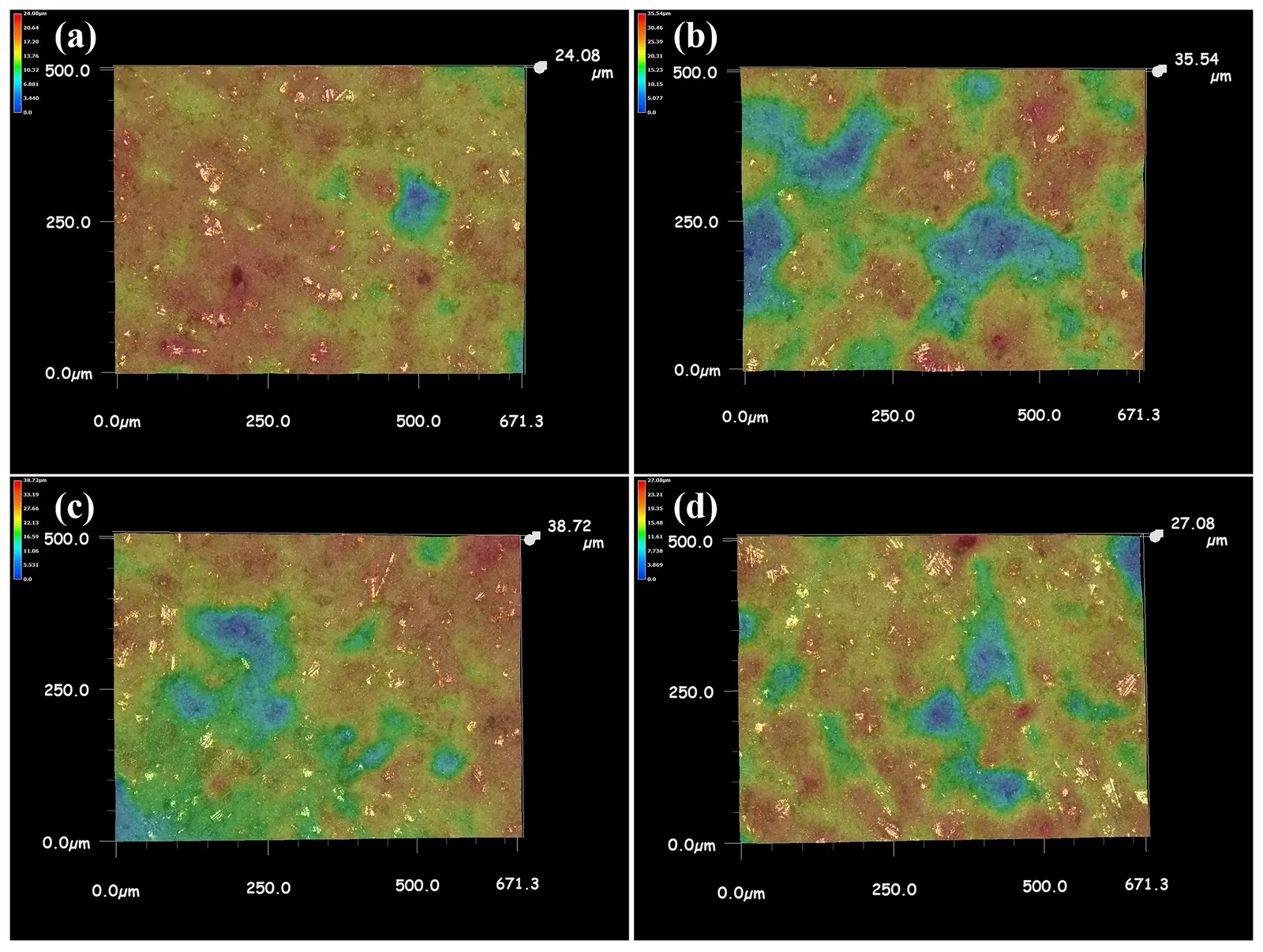
Three-dimensional surface topographies of the resin-bonded diamond tools containing different pore-forming additives: (**a**) control group, (**b**) NH_4_HCO_3_, (**c**) NaHCO_3_, and (**d**) NaCl.

To further examine the working surface features associated with differences in apparent porosity, the tool surfaces were characterized using a digital microscope with an extended depth of field, as shown in Figure 8. The field of view was 671.3 μm × 500.0 μm for all images. In each image, the color scale from red to blue denotes decreasing relative surface height. Considering the tool-fabrication process, the pronounced blue–green depressions can be interpreted as pore or associated depressions exposed at the working surface.

As shown in Figure 8a, the control tool exhibited a relatively flat surface with only a few isolated depressions, and the surface height range within the field of view was 24.08 μm. The addition of pore-forming agents produced marked changes in the number, lateral dimensions, morphology, and spatial distribution of pores on the working surface. The tool containing NH_4_HCO_3_ exhibited numerous irregular pores distributed over a wider area, with some adjacent depressions appearing to merge locally. Its surface height range increased to 35.54 μm (Figure 8b). The tool containing NaHCO_3_ exhibited relatively large, irregular depressions concentrated in localized regions, indicating pronounced clustering. Its surface height range reached 38.72 μm, the largest among the four groups (Figure 8c). Several elongated or irregular depressions were also observed in the NaCl group, although their number and areal coverage were generally lower than those in the NH_4_HCO_3_ group. The corresponding surface height range was 27.08 μm (Figure 8d). These morphological observations were broadly consistent with the trends in apparent porosity, indicating that the pore-forming agents altered both the overall porosity level and the morphology and spatial distribution of pores available at the lapping interface.

At the lapping interface, pores interrupt otherwise continuous, dense contact regions and provide space to accommodate silicon debris, binder debris, and fractured abrasive grains. They also facilitate the retention and replenishment of lapping fluid within the contact zone. These functions can reduce tool loading and surface glazing while helping to maintain diamond grain exposure. However, the beneficial effects of pores do not necessarily increase monotonically with apparent porosity or surface height range. Excessively large or locally clustered pores can reduce the effective load-bearing area of the binder and concentrate the load in the surrounding solid regions. This may weaken the support provided by the binder to the abrasive grains and increase the risk of premature grain pull-out. A relatively uniform distribution of suitably sized pores can therefore help balance debris accommodation and structural load-bearing capacity.

The NH_4_HCO_3_ and NaHCO_3_ groups had similar apparent porosities but exhibited distinctly different distributions of surface pores. In the NH_4_HCO_3_ group, the openings were distributed more widely across the field of view, providing dispersed spaces for debris accommodation at the lapping interface. Although the NaHCO_3_ group exhibited a greater surface height range, its larger depressions were concentrated in localized regions, potentially leading to spatial variations in load support and debris evacuation. These differences illustrate that apparent porosity reflects the overall volume fraction of open pores but does not fully describe their functional contribution at the lapping interface. The observed morphological differences also provide a plausible explanation for the different MRR values of the two tools despite their similar apparent porosities. The results therefore suggest that material-removal behavior is jointly governed by apparent porosity and the size, morphology, and spatial distribution of pores on the working surface, rather than by apparent porosity alone.

The combined apparent porosity, three-dimensional surface topography, and MRR results indicate that NH_4_HCO_3_ produced the highest apparent porosity without evident solid residues and generated numerous pores distributed widely across the working surface. These features helped the tool maintain relatively high and stable material removal during the early and intermediate stages of lapping. NH_4_HCO_3_ therefore exhibited the best overall pore-forming performance among the three agents investigated.

### 3.4. Orthogonal Optimization of Modifying Additive Contents

#### 3.4.1. Orthogonal Experimental Results

The preceding single-factor experiments demonstrated that PTFE, NH_4_HCO_3_, and CeO_2_ exhibited favorable performance in the resin-bonded diamond lapping tool system. To investigate the combined effects of different modifying additives on the performance of the lapping tools and the lapping performance of single-crystal silicon wafers, an orthogonal experiment was conducted using lapping tools containing different contents of PTFE, CeO_2_, and NH_4_HCO_3_. The effects of these additive contents on the surface roughness Sa, material-removal rate (MRR), and material removal-to-tool wear ratio (W-ratio) were systematically evaluated.

**Figure 9:**
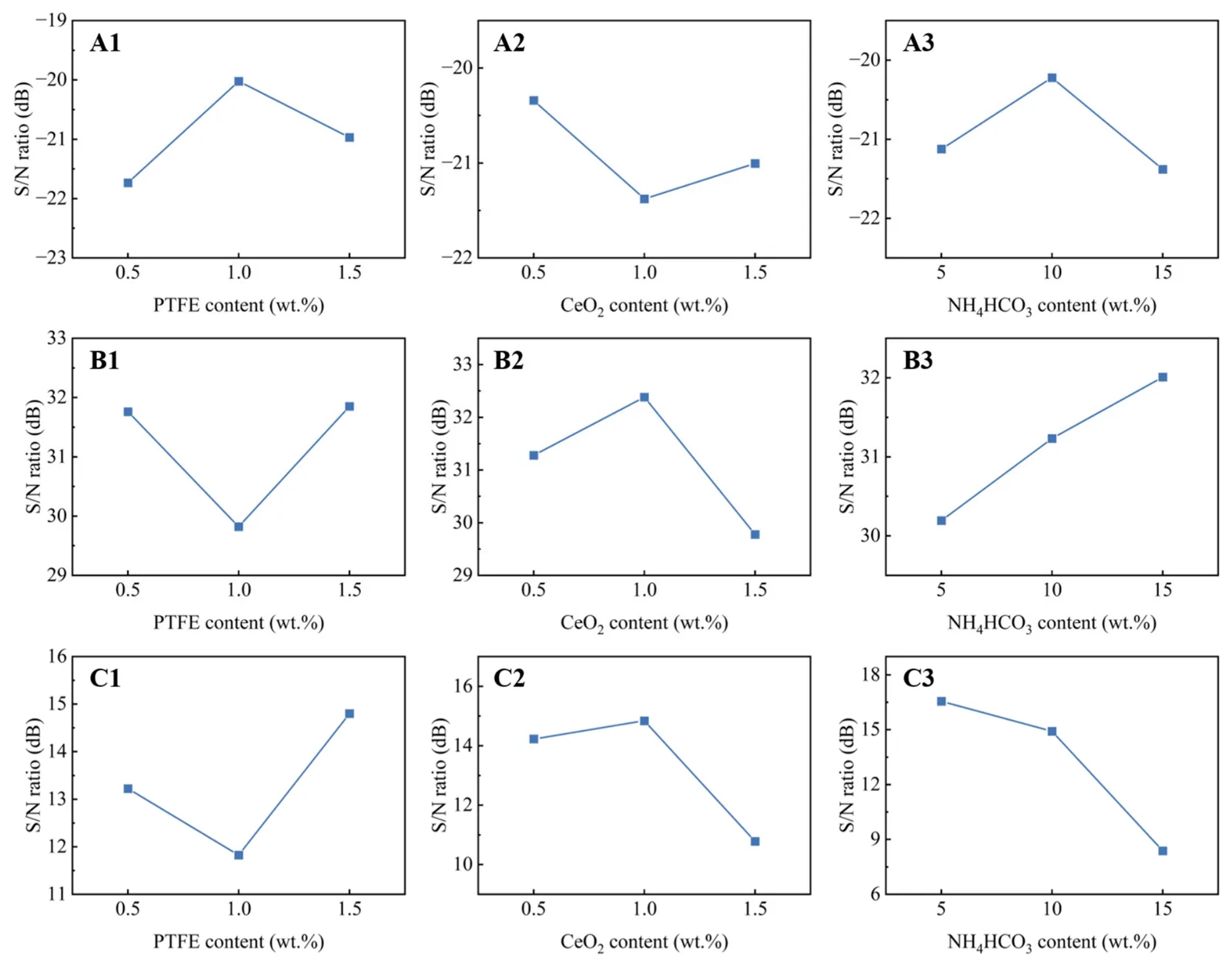
Main effects of modifying-additive contents on the S/N ratios of (**A1**–**A3**) Sa, (**B1**–**B3**) MRR, and (**C1**–**C3**) W-ratio: (**A1**–**C1**) PTFE, (**A2**–**C2**) CeO_2_, and (**A3**–**C3**) NH_4_HCO_3_.

The additive levels used in the orthogonal experimental design are listed in Table 4. Each of the nine factor-level combinations was evaluated in five independent lapping runs, resulting in a total of 45 runs. The results for each combination are presented in Table 5 as the mean ± standard deviation of the five independent runs. To evaluate the effects of the different levels of each modifying additive on the experimental responses, the signal-to-noise (S/N) ratio was employed. For Sa, the S/N ratio was calculated according to the smaller-the-better criterion, whereas the larger-the-better criterion was adopted for MRR and W-ratio. The main effects of the three factors on the S/N ratios of Sa, MRR, and W-ratio are shown in Figure 9.

#### 3.4.2. S/N Ratio and ANOVA Analyses

1. Mean S/N Ratio Response Analysis

Figure 9(A1–A3) shows the effects of PTFE, CeO_2_, and NH_4_HCO_3_ contents on the S/N ratio of surface roughness Sa. As the PTFE content increased from 0.5 wt.% to 1.0 wt.%, the S/N ratio of Sa increased from −21.73 to −20.02 dB. When the PTFE content was further increased to 1.5 wt.%, the S/N ratio decreased to −20.97 dB. Therefore, 1.0 wt.% PTFE was identified as the optimum level for minimizing Sa. An appropriate PTFE content can improve the lubrication state at the interface between the lapping tool and the silicon wafer, thereby reducing friction, adhesion, and instability of abrasive motion and consequently suppressing deep scratches and brittle damage. However, excessive PTFE may alter the mechanical properties of the resin binder and the effective protrusion of diamond abrasives, resulting in deterioration of the machined surface quality.

As the CeO_2_ content increased from 0.5 wt.% to 1.0 wt.%, the S/N ratio of Sa decreased from −20.34 to −21.38 dB, and then increased to −21.01 dB when the CeO_2_ content was further increased to 1.5 wt.%. These results indicate that 0.5 wt.% CeO_2_ is more favorable for achieving a lower and more stable surface roughness. At a relatively low content, CeO_2_ may provide moderate chemical assistance at the silicon surface, facilitating uniform removal of the surface reaction layer. With increasing CeO_2_ content, however, particle agglomeration or local accumulation may lead to nonuniform material removal and consequently degrade the surface quality.

When the NH_4_HCO_3_ content increased from 5 wt.% to 10 wt.%, the S/N ratio of Sa increased from −21.12 to −20.22 dB. A further increase to 15 wt.% caused the S/N ratio to decrease to −21.38 dB. Therefore, 10 wt.% NH_4_HCO_3_ was determined to be the optimum level for reducing Sa. An appropriate pore content can improve chip accommodation and debris evacuation within the lapping tool and reduce secondary scratching caused by retained debris. In contrast, excessive pore-forming agent may result in a loose tool structure and reduced support stiffness for the abrasive grains, thereby decreasing the uniformity of the machined surface.

Figure 9(B1–B3) shows the effects of the three modifying additive contents on the S/N ratio of MRR. The S/N ratio of MRR first decreased and then increased with increasing PTFE content. The highest S/N ratio was obtained at 1.5 wt.% PTFE; however, the difference between the 1.5 wt.% and 0.5 wt.% levels was only 0.09 dB, indicating that the material-removal performance at these two levels was comparable, whereas the MRR at 1.0 wt.% PTFE was relatively low.

As the CeO_2_ content increased from 0.5 wt.% to 1.0 wt.%, the S/N ratio of MRR increased from 31.28 to 32.38 dB. When the CeO_2_ content was further increased to 1.5 wt.%, the S/N ratio markedly decreased to 29.78 dB. These results indicate that 1.0 wt.% CeO_2_ can provide a more favorable chemical–mechanical synergistic effect, promoting the formation of a surface reaction layer on the silicon wafer and its subsequent mechanical removal. Excessive CeO_2_ may agglomerate or accumulate on the working surface of the lapping tool, thereby hindering the effective cutting action of diamond abrasives and reducing the material-removal efficiency.

Increasing the NH_4_HCO_3_ content continuously promoted the S/N ratio of MRR. As the NH_4_HCO_3_ content increased from 5 wt.% to 15 wt.%, the S/N ratio increased from 30.19 to 32.01 dB. Increasing the pore-forming agent content can increase the number of pores and the degree of abrasive protrusion, improve chip accommodation and debris evacuation, and promote self-sharpening of the lapping tool, thereby enhancing material-removal efficiency.

Figure 9(C1–C3) shows the effects of the three modifying additive contents on the S/N ratio of the W-ratio. As the PTFE content increased from 0.5 wt.% to 1.0 wt.%, the S/N ratio of the W-ratio decreased from 13.22 to 11.82 dB. When the PTFE content was further increased to 1.5 wt.%, the S/N ratio increased to 14.80 dB. Therefore, 1.5 wt.% PTFE exhibited the highest S/N ratio of the W-ratio, indicating that this content was favorable for improving the overall wear resistance of the lapping tool.

At CeO_2_ contents of 0.5 and 1.0 wt.%, relatively high S/N ratios of the W-ratio were obtained, with only a small difference between the two levels. When the CeO_2_ content was increased to 1.5 wt.%, the S/N ratio decreased to 10.78 dB. This suggests that an appropriate CeO_2_ content can facilitate material removal from the silicon surface while limiting tool wear to a certain extent. However, excessive CeO_2_ may accelerate wear of the working layer or adversely affect the ability of the resin binder to retain the diamond abrasives.

As the NH_4_HCO_3_ content increased from 5 wt.% to 15 wt.%, the S/N ratio of the W-ratio continuously decreased from 16.56 to 8.37 dB, indicating that increasing the porosity markedly reduced the wear resistance of the lapping tool. Although higher porosity can improve chip accommodation and self-sharpening, excessive pores weaken the structural integrity of the lapping tool and reduce the support and retention of diamond abrasives by the resin binder. This promotes premature abrasive pull-out and accelerates tool wear, ultimately resulting in a lower W-ratio.

2. Analysis of Variance

Analysis of variance (ANOVA) was employed within the Taguchi method to evaluate the relative influence of each factor on the responses obtained from the orthogonal experiments. ANOVA quantifies the variation associated with each factor based on the corresponding sum of squares [36]. The total corrected sum of squares, SST, is equal to the sum of the treatment sums of squares for all factors, SS_k_, and the error sum of squares, SS_e_, as expressed in Equation (7):

SS_T_ = SS_A_ + SS_B_ + SS_C_ + SS_e_ (7)

The analysis of the nine combinations in the orthogonal design had a total of eight degrees of freedom. Each of the three factors, with three levels per factor, accounted for two degrees of freedom, leaving two degrees of freedom for the residual. Interaction terms were not explicitly included in the model; however, this does not imply an a priori assumption that interactions were absent. Unmodeled interactions may be confounded with the main effects or partially absorbed into the residual term. Accordingly, the residual terms reported in Table 6, Table 7 and Table 8 represent the pooled residual variation under the additive main-effects model and cannot be regarded as pure error arising solely from random experimental variation.

For S_a_, PTFE content had the largest percentage contribution, at 49.44% (*F* = 7.09, *p* = 0.12), followed by NH_4_HCO_3_ at 24.93% (*F* = 3.57, *p* = 0.22) and CeO_2_ at 18.65% (*F* = 2.67, *p* = 0.27). Although none of the three factors had a statistically significant effect on S_a_ at the 0.05 level, the contribution percentages indicated that PTFE content had the largest estimated relative influence on S_a_ within the investigated range. This trend may be associated with the role of PTFE in regulating friction and adhesion at the tool–wafer interface. An appropriate PTFE content can help stabilize abrasive motion, thereby reducing deep scratches and brittle damage. NH_4_HCO_3_ may affect surface quality by modifying the tool pore structure, debris accommodation and evacuation, and abrasive grain exposure. By comparison, CeO_2_ content made a smaller contribution to variations in S_a_, suggesting that its potential chemical assistance may not be the primary factor governing S_a_ within the concentration range examined.

For MRR, CeO_2_ content had the largest percentage contribution, at 42.21% (*F* = 8.78, *p* = 0.10), followed by PTFE at 32.47% (*F* = 6.76, *p* = 0.13) and NH_4_HCO_3_ at 20.51% (*F* = 4.27, *p* = 0.19). None of these effects reached statistical significance at the 0.05 level, although CeO_2_ exhibited the largest estimated relative influence. This trend may reflect the participation of CeO_2_ in interfacial chemical reactions that promote the formation of a more readily removable reaction layer on the silicon surface, thereby facilitating mechanical material removal. The contribution of PTFE suggests that interfacial lubrication may influence both the effective contact between the abrasive grains and the wafer and the resulting material-removal behavior. Pores generated by NH_4_HCO_3_ may affect MRR by improving debris accommodation and evacuation and promoting abrasive grain exposure, although its relative contribution was lower than those of CeO_2_ and PTFE.

For the W-ratio, NH_4_HCO_3_ content contributed 67.00% (*F* = 8.44, *p* = 0.11), considerably more than CeO_2_ at 17.14% (*F* = 2.16, *p* = 0.32) and PTFE at 7.93% (*F* = 1.00, *p* = 0.50). Although none of these effects was statistically significant at the 0.05 level, both the contribution percentages and the S/N response trends indicated that NH_4_HCO_3_ content had the largest relative influence on the W-ratio. Increasing the NH_4_HCO_3_ content increased the number of pores within the tool. While this can improve debris accommodation and self-sharpening, it may also weaken the structural framework of the tool and reduce the ability of the resin binder to retain diamond grains. The resulting increase in tool wear could explain the decreasing trend in the W-ratio. CeO_2_ may influence the W-ratio by altering the balance between workpiece material removal and tool wear, whereas PTFE had the smallest relative influence within the investigated range.

Because the residual term in the L_9_ orthogonal design had only two degrees of freedom, the statistical power of the *F*-tests was limited. The contribution percentages and S/N response trends were therefore used primarily to compare the relative influences of the factors, rather than as evidence of statistically significant effects. PTFE, CeO_2_, and NH_4_HCO_3_ exhibited the largest relative contributions to S_a_, MRR, and the W-ratio, respectively. These findings suggest that the three classes of additives may influence lapping performance through different mechanisms, including interfacial lubrication, chemical–mechanical synergy, and pore-structure control.

When S_a_, MRR, and the W-ratio were considered individually, the preferred combinations identified by the S/N responses were A_2_B_1_C_2_, A_3_B_2_C_3_, and A_3_B_2_C_1_, respectively. With MRR and the W-ratio as the primary selection criteria, levels A_3_ and B_2_ were favored because they were common to the preferred combinations for both responses, suggesting that these levels could help balance material-removal efficiency and tool-wear resistance. Compared with A_2_, A_3_ reduced the S/N ratio for S_a_ by 0.95 dB but increased those for MRR and the W-ratio by approximately 2.03 and 2.98 dB, respectively. For NH_4_HCO_3_, increasing the content from C_2_ to C_3_ provided only a further 0.78 dB increase in the S/N ratio for MRR, whereas the S/N ratio for S_a_ decreased by 1.16 dB and that for the W-ratio dropped markedly from 14.92 to 8.37 dB. Level C_2_ was therefore considered a practical compromise among the three responses. Based on these shared preferred levels and the incremental performance gains and losses, A_3_B_2_C_2_, comprising 1.5 wt.% PTFE, 1.0 wt.% CeO_2_, and 10 wt.% NH_4_HCO_3_, was selected as the candidate balanced formulation for validation testing. This formulation represents an engineering compromise within the investigated factor ranges and under the additive main-effects model, rather than a unique optimum established through statistical significance or a weighted objective function.

#### 3.4.3. Validation of the Machining Performance of the Balanced Formulation

Based on the results of the S/N ratio analysis and ANOVA, A_3_B_2_C_2_ was selected as the balanced formulation in terms of overall performance. The corresponding contents of PTFE, CeO_2_, and NH_4_HCO_3_ were 1.5 wt.%, 1.0 wt.%, and 10 wt.%, respectively. A validation lapping experiment was subsequently conducted using the same processing conditions as those employed in the orthogonal experiments.

Table 9 summarizes the machining results obtained in the validation experiment. The Sa of the silicon wafer lapped using the A_3_B_2_C_2_ lapping tool was 8.46 nm, which was 6.00% lower than the minimum value obtained in the orthogonal experiments. The MRR reached 58.85 nm/min, representing an increase of 23.56% compared with the highest value obtained in the orthogonal experiments. In contrast to Sa and MRR, the W-ratio of the A_3_B_2_C_2_ formulation was 7.67, which was 17.88% lower than the maximum value of 9.34 obtained in the orthogonal experiments. These results indicate that the A_3_B_2_C_2_ combination did not simultaneously optimize all three individual responses, but instead achieved a multi-response balance among surface quality, material-removal efficiency, and tool durability. Under the relatively high MRR condition, abrasive dulling, fracture, and pull-out, together with wear of the binder matrix, may be accelerated, thereby increasing lapping-tool consumption and resulting in a W-ratio lower than the single-response optimum obtained in the orthogonal experiments. Therefore, the improvement in MRR achieved by the A_3_B_2_C_2_ formulation was accompanied, to some extent, by a reduction in tool durability.

**Figure 10:**
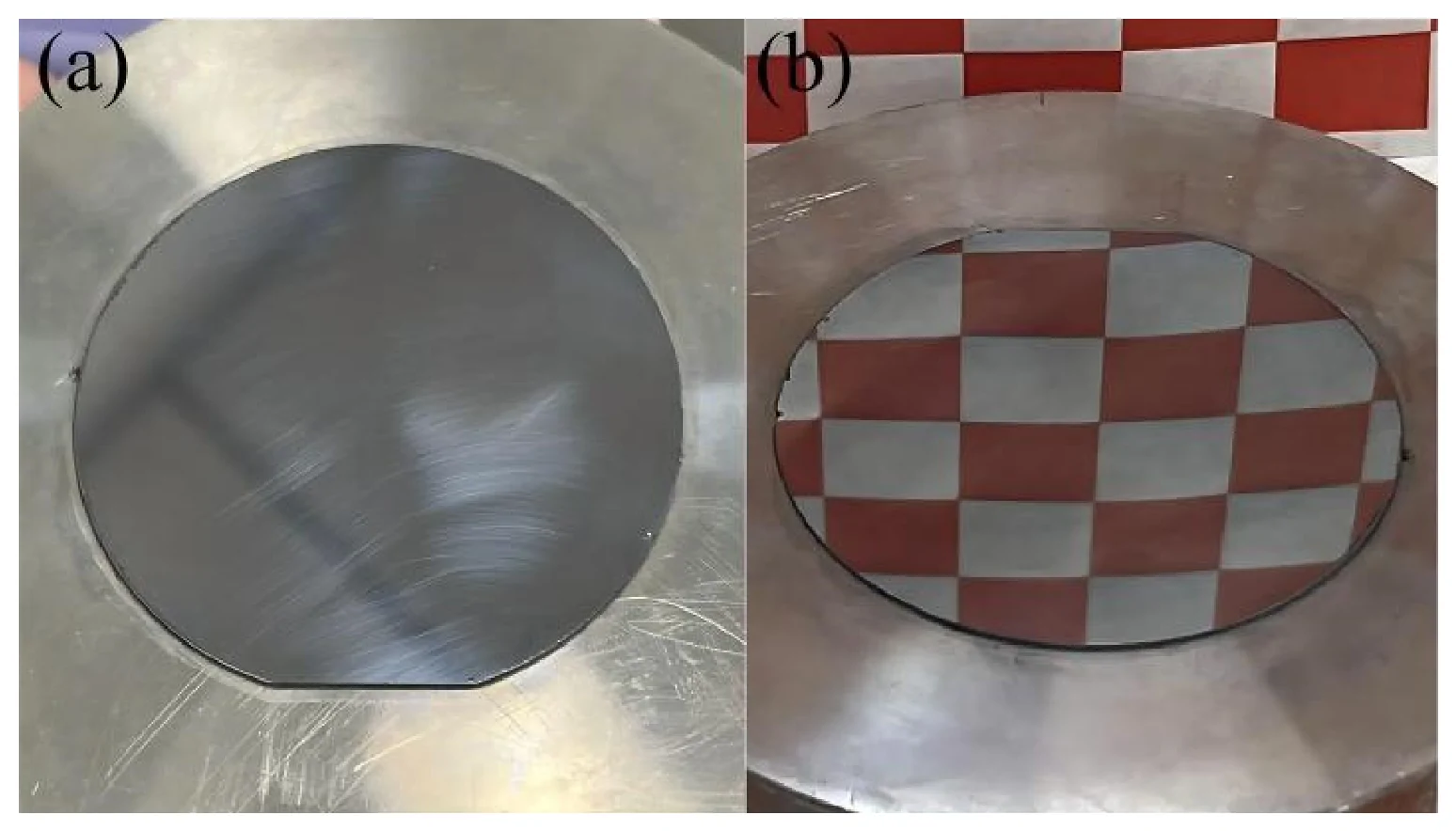
Surface of the single-crystal silicon wafer after lapping with the balanced A_3_B_2_C_2_ tool (**a**) machining textures and (**b**) mirror-like surface.

**Figure 11:**
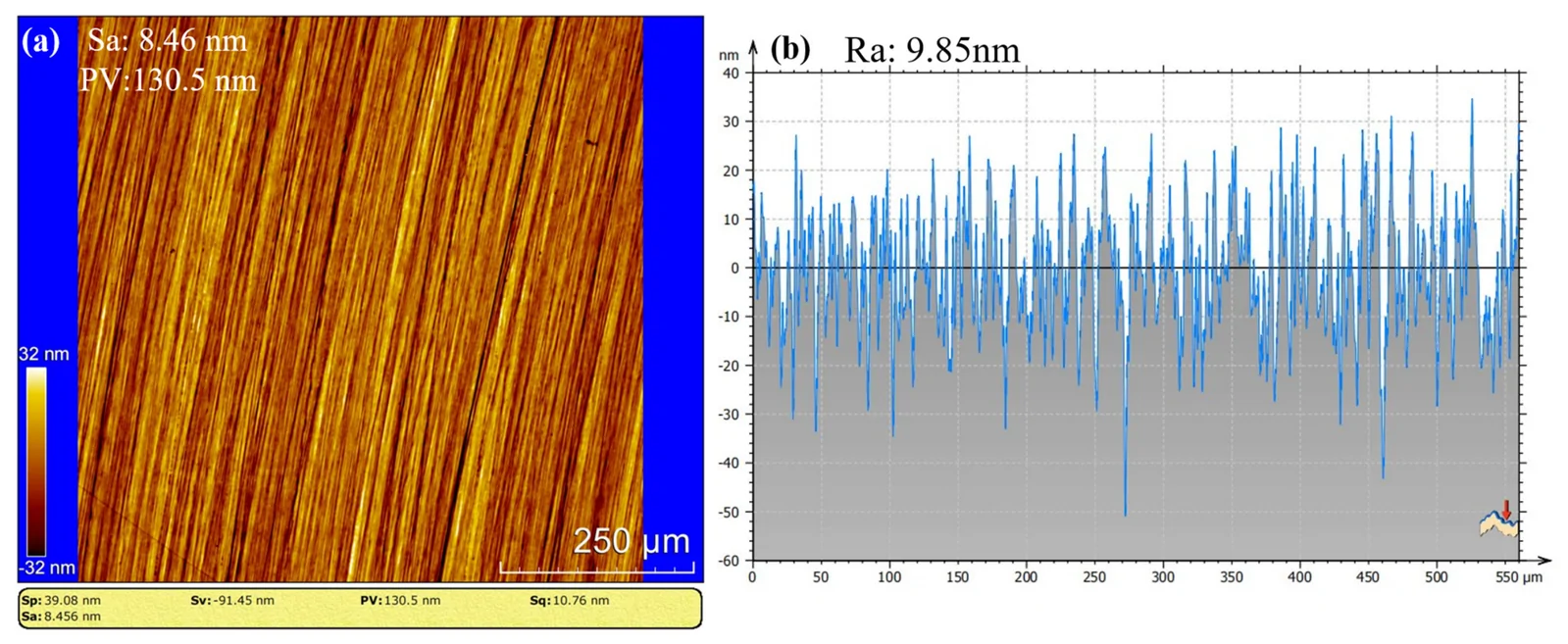
(**a**) White-light interferometric surface topography and (**b**) surface profile of the silicon wafer lapped using the balanced A_3_B_2_C_2_ tool.

**Figure 12:**
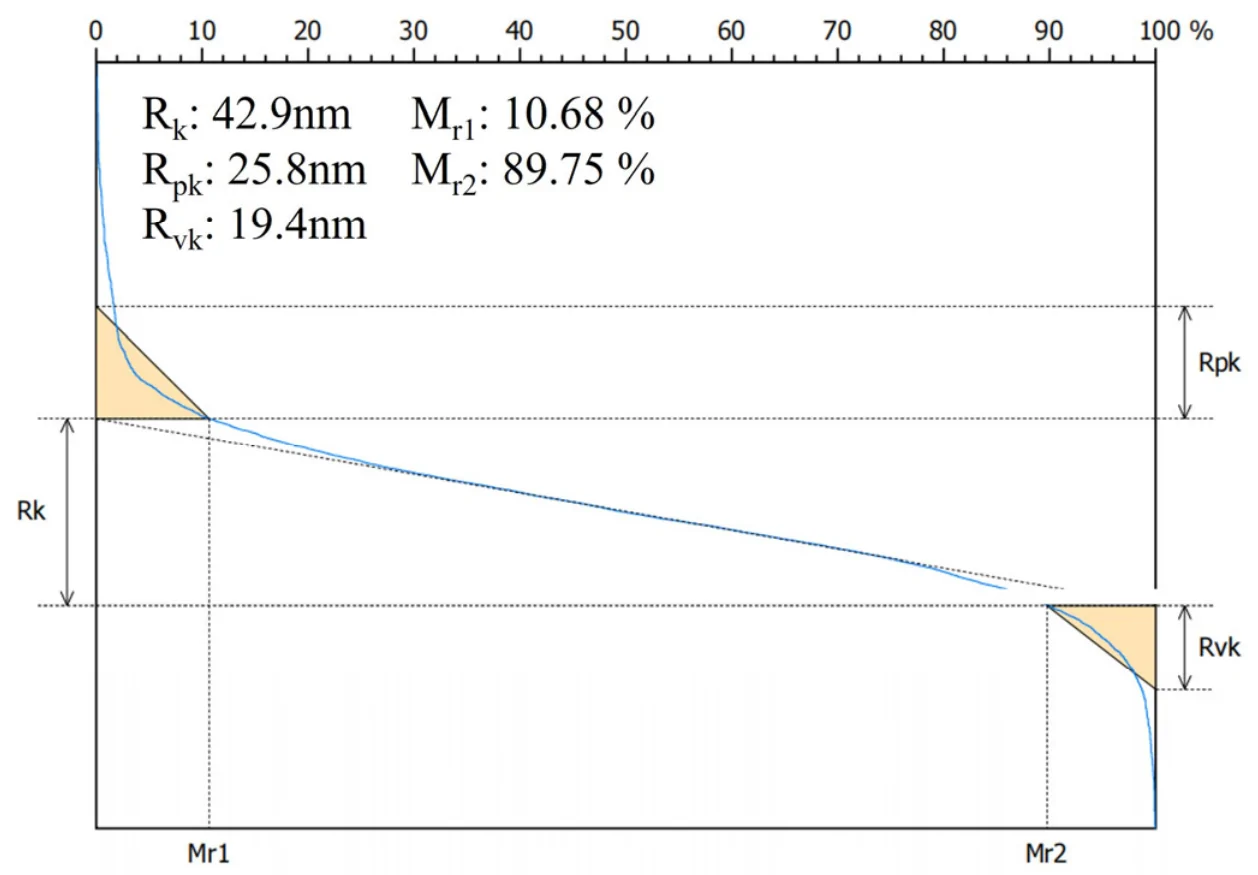
Abbott–Firestone material ratio curve of the single-crystal silicon surface processed with the A_3_B_2_C_2_ balanced-formulation tool.

The surface of the single-crystal silicon wafer lapped using the A_3_B_2_C_2_ lapping tool exhibited distinct machining textures, as shown Figure 10a, and a clear mirror-like appearance, as shown Figure 10b. Figure 11 presents the white-light interferometric topography and surface profile of the silicon wafer machined using the A_3_B_2_C_2_ lapping tool. As shown in Figure 11a, the machined surface was mainly characterized by fine strip-like textures aligned along the direction of relative motion, with relatively uniform widths and distributions. Within the observed area, no obvious deep scratches, local brittle spalling, fracture pits, or other macroscopic machining defects were observed, indicating that the lapping tool provided relatively stable material removal. The Sa and PV measured by white-light interferometry were 8.46 nm and 130.5 nm, respectively. The Ra measured using the surface profilometer was 9.85 nm, as shown in Figure 11b. As shown in Figure 12, the surface lapped using the tool prepared with the balanced formulation exhibited a core roughness depth (*R_k_*) of 42.9 nm. The reduced peak height (*R_pk_*) and reduced valley depth (*R_vk_*) were 25.8 and 19.4 nm, respectively. The material ratios *Mr*_1_ and *Mr*_2_ were 10.68% and 89.75%, respectively, indicating that the core region spanned approximately 79.07% of the material ratio range. The material ratio curve exhibited a smooth overall transition, with the peak and valley regions occupying similar proportions of the range. These features suggest that surface heights were concentrated primarily within the core region, with a limited proportion of localized protruding peaks and deep valleys. The slightly higher *R_pk_* compared with *R_vk_* suggests that peak features were relatively more pronounced.

The profile curve fluctuated relatively uniformly around the reference line, which was consistent with the uniformly distributed fine lapping textures observed in the white-light interferometric topography. These results collectively demonstrate that the A_3_B_2_C_2_ lapping tool was capable of producing a machined surface with nanometer-scale roughness.

**Figure 13:**
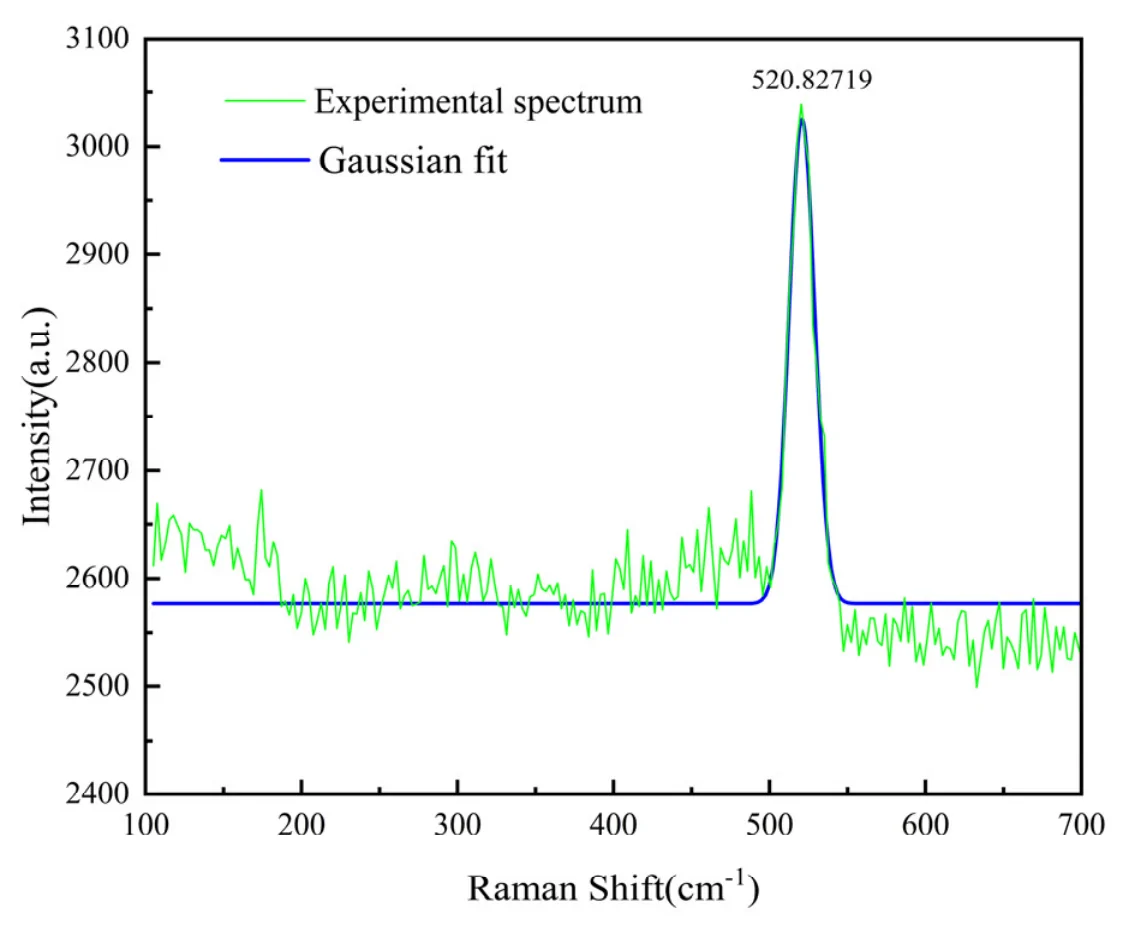
Raman spectrum and Gaussian fitting result of the silicon wafer lapped using the balanced A_3_B_2_C_2_ tool.

Figure 13 shows the Raman spectrum and corresponding Gaussian fitting result of the lapped single-crystal silicon surface. The residual stress was approximately −55.20 MPa. This value was substantially lower in magnitude than that of the control group without a surface-quality modifier and its magnitude was slightly lower than that obtained using the lapping tool containing 1 wt.% CeO_2_ alone. Therefore, despite achieving a relatively high material-removal rate, the balanced formulation did not induce obvious accumulation of residual stress and was able to maintain a relatively low level of machining-induced residual stress.

**Figure 14:**
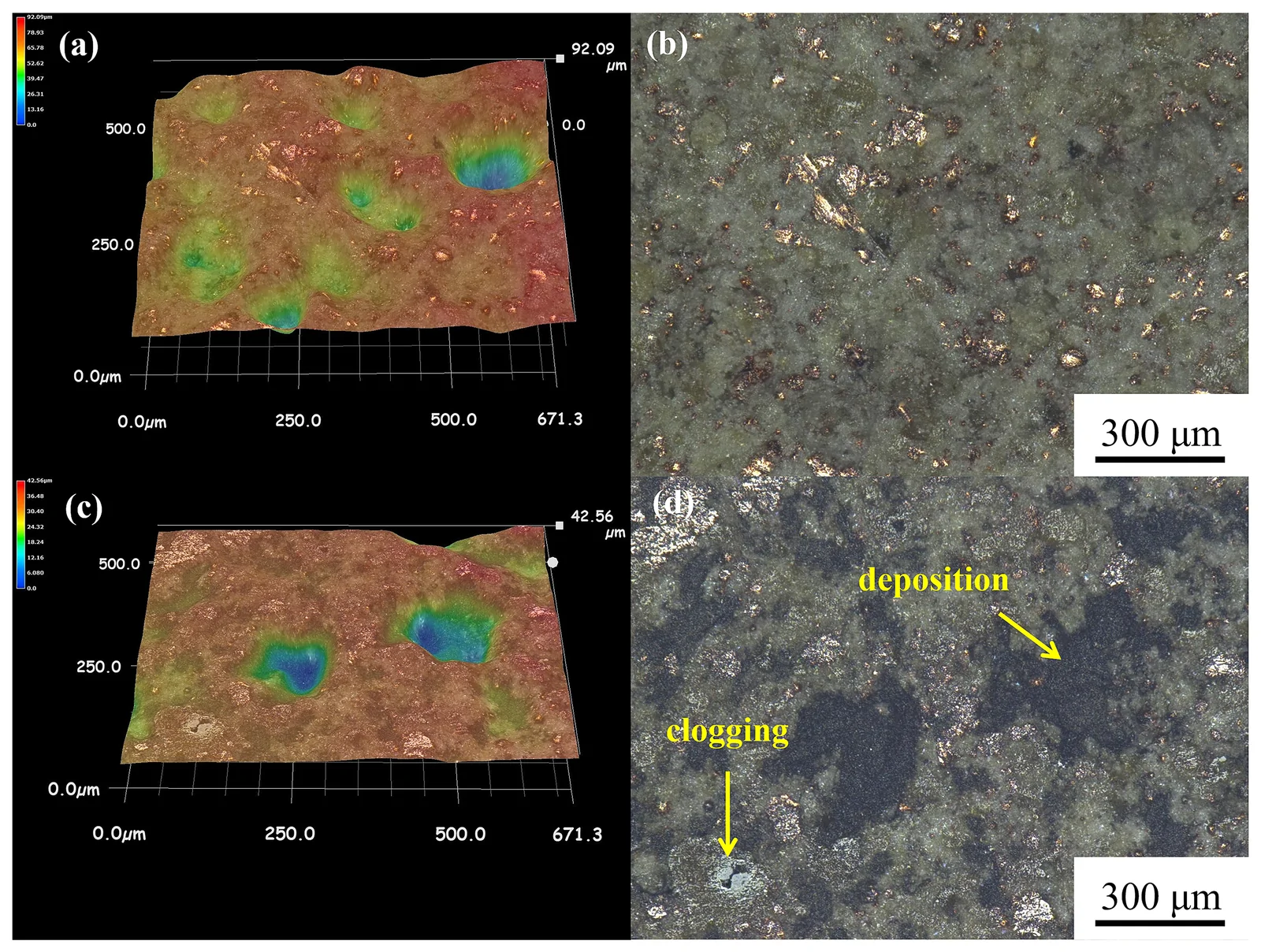
Surface topography of the balanced A_3_B_2_C_2_ lapping tool before and after lapping: (**a**,**b**) before lapping; (**c**,**d**) after lapping.

Figure 14 presents the extended-depth-of-field microscopic morphologies of the A_3_B_2_C_2_ lapping tool before and after lapping. Before use, distinct open pores were distributed over the lapping-tool surface, and the pore contours were similar to the original morphology of the pore-forming agent particles, indicating that NH_4_HCO_3_ generated pores retaining characteristics of the original particle morphology during curing. The three-dimensional reconstructed images showed that the pores were relatively deep and clean before lapping. These open pores provided accommodation space for silicon debris and abrasive fragments generated during machining and facilitated coolant transport and abrasive exposure, thereby reducing repeated abrasion by debris trapped at the lapping tool–wafer interface.

After lapping, the pores on the lapping-tool surface became noticeably shallower, and considerable dark deposits together with a small amount of adhered debris were observed within and around the pores. Continuous covering layers were also formed in some regions, indicating pronounced debris deposition, pore blockage, and local glazing. During lapping, removed silicon debris, abrasive fragments, and binder debris entered the pores, thereby reducing their effective depth and chip-accommodation volume. Meanwhile, the adhered layer covered part of the cutting edges of the diamond abrasives and reduced the number of active abrasives, causing the working surface of the lapping tool to gradually evolve toward a failure state dominated by loading and local glazing.

The loading morphology of the lapping-tool surface indicates that the pore structure generated by the A_3_B_2_C_2_ formulation effectively improved chip accommodation and debris evacuation during the initial stage of lapping, thereby promoting material removal. However, with increasing lapping time, the continuous accumulation of silicon debris within the pores gradually weakened their functional effectiveness and may have increased local friction and tool wear. This observation is consistent with the relatively high MRR but non-optimal W-ratio obtained for the A_3_B_2_C_2_ formulation.

## 4. Conclusions

In this study, a resin-bonded diamond lapping tool system for single-crystal silicon was developed by incorporating solid lubricants, surface-quality modifiers, and pore-forming agents. Single-factor experiments were conducted to investigate the effects of different modifying additives on the tribological behavior and pore structure of the lapping tools, as well as on the lapping quality of single-crystal silicon. Based on these results, the contents of PTFE, CeO_2_, and NH_4_HCO_3_ were further optimized using an orthogonal experimental design. The results demonstrate that the different modifying additives affect the lapping process through regulation of interfacial friction, pore structure, and potential chemical assistance. These mechanisms interact to jointly determine the machined-surface quality, material-removal efficiency, and lapping-tool wear. The main conclusions are as follows:

1. Solid lubricants effectively regulated the frictional and thermal behavior at the lapping tool–workpiece interface, among which PTFE exhibited the best overall lubrication performance. Compared with the control group without a solid lubricant, the average coefficient of friction of the PTFE group decreased by 18.5%. Although graphite exhibited the lowest average coefficient of friction, PTFE provided a more stable frictional response during lapping and limited the wafer temperature rise after 10 min of dry lapping to 4.1 °C, which was 18.0% lower than that of the control group.
2. CeO_2_ substantially improved the lapped surface quality of single-crystal silicon and reduced machining-induced residual stress. With the addition of 1 wt.% CeO_2_, the wafer surface Sa decreased from 11.72 nm for the control group to 9.47 nm, corresponding to a reduction of 19.2%, while PV decreased from 212.1 to 121.0 nm, representing a reduction of 42.9%. The characteristic Raman peak was located at 520.85238 cm^−1^, corresponding to a residual stress of −66.13 MPa. The magnitude of the compressive residual stress was reduced by approximately 86.5% compared with the control group.
3. NH_4_HCO_3_ exhibited the best overall pore-forming performance. The lapping tool containing 10 wt.% NH_4_HCO_3_ achieved an apparent porosity of 32.42%, which was substantially higher than the 6.76% of the control group. NH_4_HCO_3_ completely decomposed during curing to generate pores within the lapping tool. The resulting porous lapping tool maintained a relatively high material-removal capability during the early and intermediate stages of lapping and exhibited a stable MRR of approximately 2.9–3.2 nm/min during the 40–60 min period.
4. The different modifying additives exhibited distinct dominant effects on surface quality, material-removal efficiency, and lapping-tool durability, and multi-response optimization yielded the A_3_B_2_C_2_ formulation. Accordingly, the A_3_B_2_C_2_ formulation consisting of 1.5 wt.% PTFE, 1.0 wt.% CeO_2_, and 10 wt.% NH_4_HCO_3_ was selected. In the validation experiment, the wafer achieved an Sa of 8.46 nm and an MRR of 58.85 nm/min, representing improvements of 6.00% and 23.56%, respectively, compared with the minimum Sa and maximum MRR obtained in the orthogonal experiments. The corresponding W-ratio was 7.67, and the residual stress of the lapped silicon surface was −55.20 MPa. A_3_B_2_C_2_ was not an extreme-value formulation optimized for a single response, but rather a balanced formulation that achieved a favorable compromise among surface quality, material-removal efficiency, and lapping-tool durability.

## Figures and Tables

**Table 1:** Basic formulation of the lapping tool.

Component	Specification	Value (wt.%)
Diamond	#8000	20
Phenolic resin	200 mesh	10
Talc powder	12,000 mesh	8
Other additives	/	Balance

**Table 2:** Average friction coefficients of lapping tools containing different solid lubricants.

Additive	Average Friction Coefficient
Control group	0.60
Graphite	0.46
MoS_2_	0.56
PTFE ^1^	0.49

^1^ Polytetrafluoroethylene.

**Table 3:** Raman fitting results and residual stresses of the lapped silicon wafers.

Additive	Raman Peak Position (cm^−1^)	*σ* (MPa)
CeO_2_	520.85238	−66.13
SiO_2_	520.89185	−83.26
ZrO_2_	521.55116	−369.40
control group	521.82925	−490.09

**Table 4:** Parameters of the orthogonal experiment for modifying additives.

Level	Factors		
	A: PTFE	B: CeO_2_	C: NH_4_HCO_3_
1	0.5%	0.5%	5%
2	1.0%	1.0%	10%
3	1.5%	1.5%	15%

**Table 5:** Results of the orthogonal experiment for modifying additives.

Run	Factors			Sa (nm)	MRR (nm/min)	W-Ratio
	A	B	C			
1	0.5%	0.5%	5%	11.71 ± 0.87	38.00 ± 8.66	9.34 ± 1.41
2	0.5%	1.0%	10%	11.46 ± 0.52	45.46 ± 11.09	7.16 ± 2.28
3	0.5%	1.5%	15%	13.51 ± 0.43	41.41 ± 11.12	2.64 ± 1.42
4	1%	0.5%	10%	9.00 ± 0.20	41.02 ± 17.27	5.99 ± 2.76
5	1%	1.0%	15%	10.94 ± 2.60	47.63 ± 16.08	4.55 ± 2.84
6	1%	1.5%	5%	9.88 ± 1.78	27.76 ± 9.61	4.63 ± 1.42
7	1.5%	0.5%	15%	10.63 ± 0.71	42.73 ± 6.69	3.36 ± 0.49
8	1.5%	1.0%	5%	12.50 ± 1.14	44.44 ± 9.50	9.29 ± 2.48
9	1.5%	1.5%	10%	10.26 ± 2.28	37.66 ± 10.23	7.58 ± 2.58

**Table 6:** Analysis of variance for Sa.

Factor	DOF	SS	SS%	*F*	*p*
A: PTFE	2	4.40	49.44	7.09	0.12
B: CeO_2_	2	1.66	18.65	2.67	0.27
C: NH_4_HCO_3_	2	2.22	24.93	3.57	0.22
D: Pooled error	2	0.62	6.98	/	/
Total	8	8.90	100	/	/

**Table 7:** Analysis of variance for MRR.

Factor	DOF	SS	SS%	*F*	*p*
A: PTFE	2	7.90	32.47	6.76	0.13
B: CeO_2_	2	10.27	42.21	8.78	0.10
C: NH_4_HCO_3_	2	4.99	20.51	4.27	0.19
D: Pooled error	2	1.17	4.81	/	/
Total	8	24.33	100	/	/

**Table 8:** Analysis of variance for W-ratio.

Factor	DOF	SS	SS%	*F*	*p*
A: PTFE	2	13.31	7.93	1.00	0.50
B: CeO_2_	2	28.78	17.14	2.16	0.32
C: NH_4_HCO_3_	2	112.49	67.00	8.44	0.11
D: Pooled error	2	13.31	7.93	/	/
Total	8	167.89	100	/	/

**Table 9:** Comparison of the measured A_3_B_2_C_2_ responses with the best values obtained in the orthogonal experiments.

Response	A_3_B_2_C_2_ Measured	Orthogonal Best	Comparison
Sa (nm)	8.46	9.00	6.00% lower
MRR (nm/min)	58.85	47.63	23.56% higher
W-ratio	7.67	9.34	17.86% lower

## Data Availability

The original contributions presented in this study are included in the article. Further inquiries can be directed to the corresponding author.
